# Beyond Co-Occurrence: A Framework for Interpreting Antibiotic–ARG Decoupling in Aquatic Systems and Its Implications for Aquatic Animal Health in a Changing World

**DOI:** 10.3390/ani16193070

**Published:** 2026-09-29

**Authors:** Dong Liu, Lingzhi Huang, Mingjun Yan, Ying Huang, Chongrui Wang, Qianqian Ku, Yaocheng Deng, Xiping Yuan

**Affiliations:** 1 Hunan Fisheries Research Institute and Aquatic Products Seed Stock Station, Hunan Academy of Agricultural Sciences, Changsha 410153, China; 2 College of Environment & Ecology, Hunan Agricultural University, Changsha 410128, China; 3 Yuelushan Laboratory, Changsha 410128, China; 4 College of Resources, Hunan Agricultural University, Changsha 410128, China

**Keywords:** antibiotics, antibiotic resistance genes, aquatic systems, evidence synthesis, chemical–genetic mismatch, legacy persistence

## Abstract

Antibiotics and antibiotic resistance genes (ARGs) are widespread in aquatic ecosystems affected by climate variability, urbanization, and intensifying aquaculture. Yet parent-antibiotic concentrations and resistance-related genetic endpoints do not always vary in parallel across seasons, sites, or environmental compartments. This discrepancy matters for aquatic animals as ecological receptors, potential vectors, and targets for antimicrobial resistance monitoring and management. To examine whether transport, retention, and biological processes may contribute to these mismatches, we conducted a structured narrative review. We screened studies retrieved from Web of Science Core Collection, Scopus, and PubMed for paired measurements in aquatic systems. Of 98 field studies, 25 provided sufficient paired data for matched chemical–genetic assessment. Among these, 16 provided Moderate evidence and 9 provided Suggestive evidence for coupled, mismatched, or compartment-dependent responses. No study met the stricter criteria for field-supported legacy persistence. Seasonal reversals, spatial divergence, and differences among water, particles, and sediments suggest that water-column antibiotic concentrations alone are incomplete proxies for ARG abundance. Such measurements alone cannot assess ARG mobility or host context. Potential contributors include sediment and biofilm retention, extracellular DNA, hydrodynamic redistribution, horizontal gene transfer, transformation products, and co-selective stressors, although their relative roles remain unresolved. These findings support monitoring that combines chemical and genetic endpoints with information on sources, hydrology, compartments, and hosts to inform aquatic-animal risk assessment and management.

## Simple Summary

As climate change and human activity reshape aquatic ecosystems, understanding how antibiotics and resistance genes behave differently matters directly for the health of aquatic animals and the safety of aquaculture products. Antibiotic concentrations and resistance-related genetic signals do not always change together in rivers, lakes, reservoirs, and estuaries. This review finds that antibiotic levels alone do not reveal how resistance-related genetic material changes. Field studies point to seasonal shifts, sediment storage, water movement, and other environmental pressures as reasons why chemical and genetic signals can diverge. Monitoring should therefore measure both antibiotics and resistance-related genetic indicators across water, sediment, and biofilms. This approach can help environmental managers distinguish continuing pollution from longer-lasting environmental effects.

## 1. Introduction

Antimicrobial resistance (AMR) poses environmental, animal-health, and public-health concerns. Aquatic environments bring together antimicrobial residues, resistant microorganisms, antibiotic resistance genes (ARGs), and mobile genetic elements (MGEs) [1,2,3]. Sulfonamides (SAs), fluoroquinolones (FQs), tetracyclines (TCs), and macrolides (MLs) are widely detected in rivers, lakes, reservoirs, estuaries, and coastal waters [4,5,6,7,8]. Major inputs include municipal wastewater, livestock production, aquaculture, pharmaceutical use, and other human activities [8,9,10]. These inputs may also introduce resistant bacteria, ARGs, MGEs, nutrients, metals, disinfectants, and other contaminants that can influence the environmental resistome.

Aquatic animals warrant attention because they inhabit environments where chemical residues and resistance determinants are transported, retained, and redistributed. Exposure may occur through surrounding water, suspended particles, sediment, diet, and animal-associated microbial communities. Antibiotics can accumulate in aquatic organisms and move through food webs, although the extent varies by compound and species [11,12,13,14]. Resistance determinants may also enter or interact with animal-associated microbiota. Experimental and field evidence indicates that aquatic organisms can contribute to the environmental dissemination of ARGs [15,16]. In aquaculture, stocking density, antimicrobial use, feed inputs, sediment accumulation, and water exchange shape these interactions [17,18,19]. Aquatic animals are therefore ecological receptors and potential carriers and monitoring targets in broader environmental and One Health assessments.

Many studies have documented the co-occurrence of antibiotics and ARGs in aquatic environments. Co-occurrence alone does not show that these endpoints share a source, environmental fate, or response trajectory. Resistance determinants predate modern antibiotic use and remain part of natural microbial communities [20,21]. Human activities can introduce resistant organisms and ARGs directly, while antibiotic residues and other stressors may promote their enrichment or selection in receiving environments [22,23,24,25,26,27,28,29,30,31,32,33]. Thus, detecting an ARG at a site with low antibiotic concentrations does not, by itself, demonstrate recent antibiotic selection or persistence following earlier exposure. Conversely, high antibiotic concentrations do not necessarily produce a proportional genetic response. Interpreting environmental antibiotic–ARG relationships requires distinguishing background resistance, external loading, environmental enrichment, in situ selection, and persistence after chemical pressure declines.

Chemical and genetic endpoints are shaped by partly distinct environmental processes. Dissolved antibiotics can be diluted, degraded, transformed, sorbed to particles, or transferred among water, sediment, and biofilm compartments. ARGs are associated with microbial cells, mobile genetic elements, extracellular DNA, and host-community dynamics. Rivers, lakes, reservoirs, and estuaries differ in residence time, particle transport, stratification, sediment–water exchange, and disturbance regimes [34,35,36,37,38,39]. An instantaneous water-column antibiotic concentration may therefore differ from the exposure history of sediment, biofilm, or animal-associated microbial communities. Seasonal runoff, floods, droughts, tidal exchange, and resuspension can redistribute both chemical and genetic material [36,37,40,41]. Heavy metals, disinfectants, microplastics, and other stressors may also influence resistance dynamics when concentrations of the measured parent antibiotic are low [22,23,24,25].

These differences matter for aquatic-animal risk assessment and management. A decrease in water-column antibiotic concentrations may indicate reduced chemical exposure, but does not necessarily mean that ARG abundance, mobility, host association, or exposure through particles, sediments, biofilms, and food webs has also declined. Likewise, a genetic signal that persists after chemical attenuation does not, by itself, establish a legacy effect. Continued source inputs, hydrological redistribution, changes in bacterial biomass, or non-antibiotic co-selection can produce similar patterns. Monitoring water-phase antibiotic concentrations alone may therefore miss changes in the biological and genetic components of AMR risk. Interpretation should combine paired chemical and genetic measurements with information on source continuity, environmental compartments, hydrological conditions, microbial hosts, and mobility.

Previous reviews have synthesized the occurrence, sources, environmental fate, removal, ecological effects, and transmission of antibiotics and ARGs in aquatic and aquaculture systems [4,7,19,42,43]. These reviews describe the role of aquatic environments in AMR dissemination. Less attention has been given to whether parent-antibiotic concentrations and resistance-related genetic endpoints change in the same direction within a field system, and how to interpret divergence between them. This distinction matters because spatial correlations or simultaneous detection cannot establish whether an ARG pattern reflects current antibiotic exposure, continued external loading, environmental retention, hydrological redistribution, or persistence after earlier chemical pressure. A synthesis focused on paired chemical–genetic responses can help distinguish observed mismatches from stronger mechanistic or temporal interpretations.

The working hypothesis is that differences in transport, retention, and biological processes can partly decouple resistance-related genetic responses from contemporaneous parent-antibiotic concentrations. We use a structured narrative evidence synthesis to examine paired measurements of antibiotics and resistance-related genetic endpoints across aquatic field studies and assess the strength of observed response patterns. We distinguish background resistomes, anthropogenic loading, environmental enrichment, selection-supported in situ amplification, and legacy persistence. These categories define the evidentiary boundaries for interpreting chemical–genetic relationships. Mechanistic evidence is used to examine candidate explanations for observed mismatches, including hydrodynamic redistribution, sediment and biofilm retention, extracellular DNA persistence, horizontal gene transfer, transformation products, and multi-stressor co-selection. Finally, we consider implications for AMR monitoring and management in aquatic animals and aquaculture, focusing on study designs that can distinguish continued loading or redistribution from genuine legacy persistence after chemical concentrations decline. Throughout, we treat aquatic animals as ecological receptors, potential vectors, and monitoring and management targets for antimicrobial resistance, and we situate the observed chemical–genetic mismatches within the broader context of climate change, urbanization, and other anthropogenic pressures that are reshaping aquatic ecosystems.

## 2. Review Methodology

This review used a structured narrative evidence-synthesis approach. Web of Science Core Collection, Scopus, and PubMed were searched from database inception to 23–24 July 2026 for studies linking parent antibiotics with antibiotic resistance genes (ARGs) and/or mobile genetic elements (MGEs) in aquatic environments. Web of Science returned 8395 records, Scopus 8235, and PubMed 4696 unique records after within-database deduplication. Complete search strings, search fields, retrieval dates, and query-level hit counts are provided in Supplementary Table S1.

**Figure 1:**
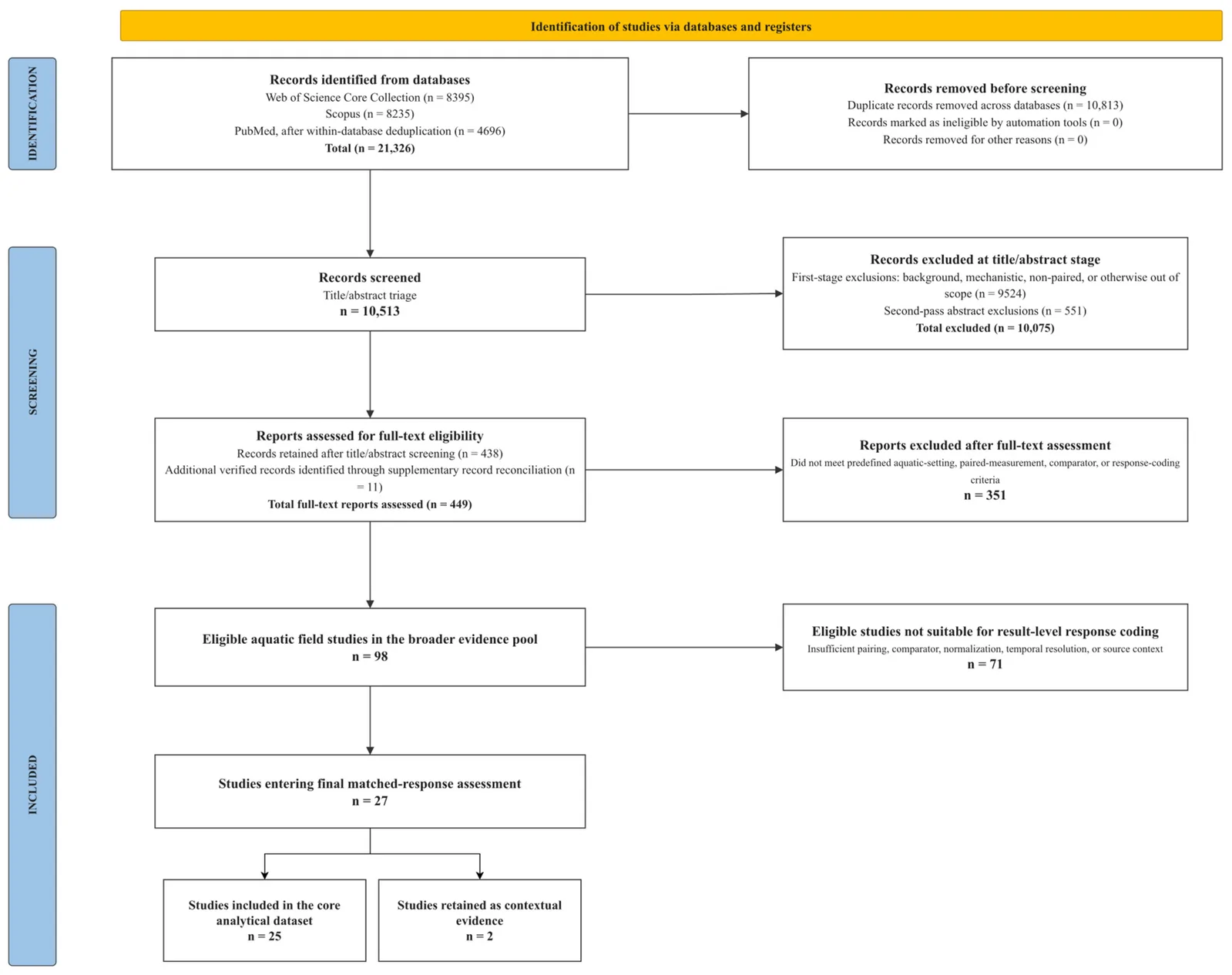
PRISMA 2020-style flow diagram for study identification and selection in the structured evidence synthesis. Notes: Records were deduplicated using DOI and, where DOI was unavailable, normalized exact-title matching. Title/abstract triage was used to prioritize manual review and did not itself establish final study eligibility. No meta-analysis was undertaken.

Across databases, 21,326 records were identified. Duplicates were removed by DOI and, where DOI was unavailable, normalized exact-title matching, leaving 10,513 records for two-stage title/abstract screening. After screening, 438 candidates remained; 11 additional verified records were added during supplementary record reconciliation, giving 449 reports for full-text assessment. Study selection was applied sequentially. First, studies entered the broader field-evidence pool only when they (1) investigated a natural or semi-natural aquatic system and (2) reported parent-antibiotic measurements together with ARG and/or MGE endpoints within the same study. Second, entry into core matched-response coding additionally required (3) chemical and genetic observations that could be aligned by sampling site, period, event, intervention, or environmental compartment; (4) a study-supported within-study comparator or reference condition; and (5) sufficient result-level information to interpret the directions of the chemical and genetic responses without constructing a new comparison not supported by the original study design. Studies that met the broader field-evidence criteria but did not meet one or more of these core requirements were not used for core response coding; they were retained either for evidence-map coverage or, when directly informative to interpretation, as contextual evidence. Of the 449 reports, 351 were excluded, leaving 98 eligible field studies. Twenty-seven had sufficient information for final matched-response assessment: 25 entered the core analytical dataset and two were retained as contextual evidence; the remaining 71 were retained for evidence-map coverage only. The selection process is summarized in Figure 1.

For the 25 core studies, we extracted study setting and design, environmental matrix, antibiotic and ARG/MGE endpoints, response direction, normalization and comparator information, and relevant hydrological, source, and environmental context. In this review, an endpoint refers to a measured or derived variable used to characterize the chemical or resistance-related genetic state of a sample or system. Chemical endpoints include parent-antibiotic concentrations or loads, whereas genetic endpoints include ARG abundance or normalized abundance, expression, association with mobile genetic elements, host linkage, and transfer-related measures. Evidence grades reflected confidence in the interpretability of the observed paired chemical–genetic response rather than overall study quality, causal attribution, or evidence for legacy persistence. The assessment considered paired measurement, reference conditions, endpoint normalization, matrix comparability, temporal or spatial resolution, and source attribution and hydrological context (Table 1). Moderate evidence indicated a directly interpretable paired response without a major comparability or resolution limitation, whereas Suggestive evidence indicated an informative pattern for which one or more limitations materially constrained interpretation. Legacy persistence was evaluated separately using a stricter criterion requiring a documented and sustained decline in antibiotic input or chemical selection pressure, temporally ordered paired chemical–genetic observations, appropriate normalization, and consideration of continued loading or hydrological redistribution.

Because study matrices, analytical methods, units, genetic targets, and reporting denominators were heterogeneous, no meta-analysis or pooled effect-size calculation was undertaken. The synthesis therefore emphasized within-study response direction, environmental context, and evidence strength. Mechanistic studies without paired field trajectories were used only to interpret candidate pathways. Study-level coding for the 25 core studies is provided in Supplementary Table S2.

## 3. Conceptual and Interpretive Framework for Antibiotic–ARG Decoupling

ARG trajectories can differ from those of the antibiotics with which they co-occur, are transported, or are selected [44,45,46,47]. Their interpretation requires a defensible reference condition, paired chemical and genetic observations, and attention to source continuity and environmental context. We therefore distinguish forms of anthropogenic influence and define the conceptual states used to interpret chemical–genetic response patterns; the operational search, selection, coding, and grading procedures are described in Section 2.

### 3.1. Natural Background Resistome and Anthropogenic Influence

Recent antibiotic selection requires evidence beyond ARG occurrence alone. Resistance determinants predate modern antibiotic use and occur in microbial communities with little identifiable human influence [20,21]. Genes annotated as resistance determinants may also have broader ecological functions, including metabolite transport, cellular defense, signaling, and microbial competition [21]. Detection in water, sediment, or biofilm establishes the presence of a resistome. Attribution to pollution, contemporary selection, or direct human-health risk requires additional evidence on enrichment, mobility, host context, and source association [26,27,28,29,30,31].

Aquatic resistomes combine this natural background with organisms and genes introduced or altered by human activity. Separating these components requires evidence on source origin, host identity, mobility, and environmental selection. A downstream increase in ARGs may reflect passive loading and survival of fecal bacteria rather than selection within the receiving water [26,27,28]. Conversely, a low total ARG abundance may still be consequential when a clinically relevant determinant occurs in a potential pathogen or on a mobile genetic element (MGE) [29,30,31]. Anthropogenic influence is more strongly supported when several signals converge, such as enrichment relative to an upstream, low-impact, historical, or matched-control reference; association with plasmids, transposons, or class 1 integrons; expanded host range or occurrence in potential pathogens; and transcriptional or transfer activity [26,27,28,29,30,31]. These signals support anthropogenic enrichment or human association. Attribution to continued loading or selection within the receiving environment requires targeted source and process evidence.

Loading and in situ selection make different predictions and should be evaluated separately. Wastewater, livestock production, aquaculture, and industrial discharge can directly introduce resistant organisms, ARGs, and MGEs [27,28,32,33]. Antibiotic residues and non-antibiotic stressors can then select or co-select organisms already present in the receiving environment [22,23,24,25]. These pathways often operate together, but they are not interchangeable. Fecal indicators, microbial source-tracking markers, longitudinal mass balances, and intervention designs should be considered when distinguishing continued organism input from selection-supported in situ amplification [26,27,44].

We use five terms consistently. The background resistome comprises resistance determinants for which no recent anthropogenic influence has been demonstrated. Anthropogenic loading is the direct introduction of resistant organisms, ARGs, or mobile genetic elements (MGEs) from wastewater, livestock production, aquaculture, industrial discharge, or other human-associated sources. Environmental enrichment is an increase in ARG abundance, expression, mobility, host range, or carriage in potential pathogens relative to a defensible environmental reference. It does not assign a single mechanism. Selection-supported in situ amplification is used when evidence indicates that selection, growth, or horizontal transfer in the receiving environment contributed to that enrichment. Legacy persistence is the continued elevation, activity, or mobility of resistance-related genetic endpoints after a documented and sustained decline in the relevant antibiotic input or chemical selection pressure. It is not inferred from continued ARG detection or from ARG occurrence under low measured parent-antibiotic concentrations. This terminology separates observed response patterns from causal interpretation while identifying the evidence needed to link them.

### 3.2. Definition and Evidence Hierarchy for Decoupling

We define antibiotic–ARG decoupling as a mismatch between the trajectory of parent-antibiotic exposure and that of an anthropogenically enriched resistome within a specified system, period, and environmental compartment. The genetic trajectory may involve persistence, enrichment, expression, or mobilization. This definition emphasizes matched trajectories and environmental context, rather than a single detection event.

The genetic evidence considered in this review captures four response dimensions: persistence, enrichment, expression, and mobilization. Persistence is a delayed or negligible decline in normalized ARG abundance after antibiotic concentrations or inputs decrease. Enrichment is maintenance above an upstream, low-impact, pre-disturbance, or matched-control reference. Expression is sustained transcriptional activity despite chemical attenuation or little change in DNA-level abundance. Mobilization is maintained or increased association of ARGs with plasmids, integrons, transposons, extracellular DNA available for natural transformation, a broadened host range, or transfer into potential pathogens [29,30,31,48]. Abundance describes how much genetic material is present. Expression, mobility, and host context more directly constrain whether that material is active and capable of dissemination [29,30,31,48].

A chemical–genetic mismatch is most informative when plausible alternatives are evaluated. Continuous or repeated loading, hydrological mixing, shifts in bacterial biomass, and fecal pollution can each shape apparent ARG patterns [26,27,36,44]. We therefore assessed whether antibiotics and ARGs or MGEs were measured in matched sampling units. We also assessed whether the design resolved time or compartment, whether ARG measurements were normalized, and whether hydrology, source intensity, biomass, and fecal inputs were considered.

Moderate and Suggestive grades describe confidence in the observed paired chemical–genetic response pattern. Moderate evidence captures paired measurements across time, space, or compartments that show different chemical and genetic responses, but do not resolve a post-decline temporal lag or all major alternatives. Suggestive evidence identifies a possible association or mismatch when temporal resolution, normalization, comparability, reference condition, or source attribution remains incomplete. Field-supported legacy persistence was evaluated using a separate, stricter inferential criterion. A field-supported legacy inference requires paired before–after, intervention, or sufficiently resolved temporal data from the same field system following a documented and sustained decline in the relevant external antibiotic input or chemical selection pressure. It must also include matched antibiotic and ARG or MGE measurements, appropriately normalized genetic endpoints, established temporal ordering, and evaluation of major alternative explanations. This approach separates observation from inference and assigns each study the strongest conclusion justified by its design.

Cross-compartment comparisons retained their original matrices and denominators. Water concentrations in ng/L were not numerically compared with sediment concentrations in ng/g. Similarly, ARG copies per mL were not treated as equivalent to copies per g. Where possible, we used within-study directions of change, removal fractions, partition coefficients, and mass distributions. We also used copies per 16S rRNA gene, reported estimates of copies per bacterial cell, and metagenomic abundance normalized by sequencing depth. When normalization was not possible, we described the pattern qualitatively as sediment- or particle-associated retention rather than fixed fold enrichment [44,49,50,51].

To translate mismatch patterns into testable process hypotheses, we use four system-level constructs. Chemical–genetic hysteresis denotes a path-dependent lag between chemical attenuation and a genetic response. Hydrological memory denotes storage and later remobilization of material from earlier conditions. Risk displacement denotes transfer of chemical or genetic risk among water, particles, sediment, biofilm, and hosts rather than true removal. Threshold lowering denotes the possibility that mixtures, biofilms, or co-selectors permit selection at lower parent-antibiotic concentrations than single-compound tests predict [22,23,24,25,36,37,39,52]. These constructs organize measurements and competing explanations. They are not treated as direct proof of any one mechanism in a field system.

## 4. Cross-System Evidence for Antibiotic–ARG Decoupling

### 4.1. Evidence Landscape

The 25 retained paired field studies encompassed wastewater-receiving rivers, mixed-use catchments, lakes and reservoirs, aquaculture waters, river–estuary continua, and water–sediment comparisons. The studies reported several response patterns: coupled chemical and genetic enrichment or attenuation, seasonal reversal, spatial mismatch, compartment-dependent association, hydrodynamically mixed responses, and scale-dependent loss of correlation. This variation shows that parent-antibiotic concentrations are not universal proxies for ARG abundance, mobility, or host context.

Taken together, these studies span a range of aquatic settings and geographic contexts, although the coverage is not evenly distributed. The core evidence includes sites in Asia, Europe, North America, and Africa, with a substantial proportion from China and other East Asian settings. Ecosystem coverage is broader, including wastewater-receiving rivers, mixed-use watersheds, lakes and reservoirs, estuaries, water–sediment systems, and a smaller number of aquaculture settings. Climatic contexts range from Mediterranean and subtropical systems to temperate and seasonally ice-covered environments, but the evidence base was not designed to provide balanced representation across climate zones. The framework should therefore be applied with explicit consideration of local hydrology, climate, source structure, and ecosystem characteristics.

The evidence grades indicate confidence in the observed chemical–genetic response. Sixteen studies provided Moderate evidence through paired seasonal, spatial, intervention, or compartmental comparisons. Nine provided Suggestive evidence because normalization, matrix comparability, reference conditions, or source attribution were incomplete. The studies indicate where paired monitoring is most informative and which field designs are needed to test post-decline legacy persistence directly. A concise study-level summary of the 25 core studies is provided in Table 2; expanded design context and full bibliographic details are retained in Supplementary Table S2.

Several studies reported broadly concordant chemical and genetic responses. Antibiotics and ARGs declined together through reservoir ecological-purification facilities [57]. Both endpoint families were higher near wastewater discharges or during low-flow periods in several receiving rivers [55,61,62]. Poyang Lake showed higher antibiotic concentrations and ARG abundance during the dry season [64]. The Chishui River showed dry-season maxima for antibiotics and most measured ARGs, with gene-specific exceptions [65]. Urbanized rivers [54] and sediments affected by pharmaceutical manufacturing [75] also showed coupled enrichment of antibiotics, ARGs, and MGEs.

These studies describe settings in which current loading, hydrological concentration, and chemical exposure remain aligned with genetic endpoints [54,55,61,62,64,65,75]. They provide a comparison for the mismatch patterns described below and reinforce the need for context-specific chemical–genetic interpretation (Table 3).

### 4.2. Seasonal, Spatial, and Compartmental Mismatch

The clearest seasonal mismatches occurred when antibiotic and ARG responses moved in opposite directions within the same campaign. Across two subtropical river basins, water-column antibiotics were higher in the dry season, whereas 16S-normalized ARG abundance was higher in the wet season. Sediments did not show the same contrast [56]. In the Songliao Basin, antibiotics again peaked in the dry season, whereas relative ARG abundance and culturable resistant bacteria peaked in the wet season [70]. An urban river affected by macrolide pollution showed a more complex reversal. Macrolide concentrations and macrolide–lincosamide–streptogramin resistance genes increased together along the dry-season pollution gradient. The genetic gradient reversed during the wet season [69]. Other seasonal surveys reported gene-, compound-, matrix-, or hydrodynamics-specific responses rather than one shared trajectory [53,58,66,67]. These reversals provide a candidate signature of chemical–genetic hysteresis. Seasonal contrasts alone cannot resolve whether delayed response, changing inputs, or hydrological redistribution caused the pattern. Rainfall, biomass, nutrient conditions, and MGE dynamics are therefore priorities for explanatory measurement [53,56,58,66,67,69,70].

Spatial evidence likewise shows that chemical and genetic signals can diverge across receiving systems. In a Dutch wastewater-receiving river, several ARGs remained detectable far downstream while measured antibiotics declined [72]. A Northern European receiving river showed increased ARGs in sediment-associated material when antibiotics in effluent and downstream water were at low ng/L concentrations [63]. At a broader scale, sediment antibiotic–ARG correlations observed along a short wastewater-impacted lake transect disappeared across river systems [71]. These contrasts may reflect the storage and later remobilization of material from earlier inputs (“hydrological memory”) or the redistribution of chemical or genetic risk among water, particles, sediment, biofilms, and hosts rather than true removal (“risk displacement”). Source continuity, transport, and retention measurements are needed to distinguish these alternatives.

**Figure 2:**
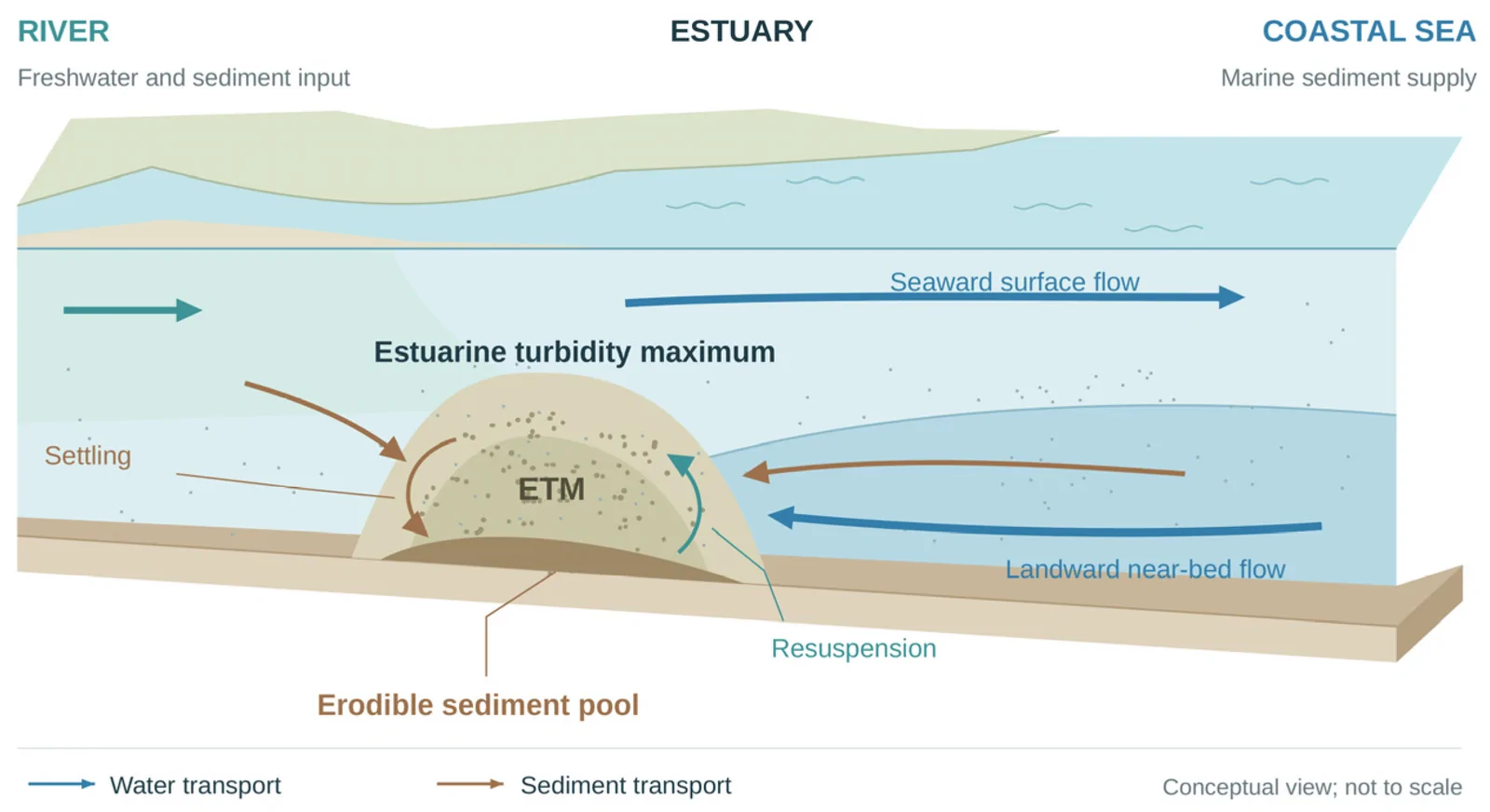
Schematic representation of suspended particulate matter (SPM) transport and trapping in an estuarine system, showing mixing intensity, water and SPM transport, estuarine circulation, the estuarine turbidity maximum (ETM), and the bottom pool of easily erodible sediment. Source: Burchard et al. [34].

Estuarine circulation provides one representative hydrodynamic setting in which suspended particulate matter (SPM) can be trapped and redistributed (Figure 2) [34].

Cross-compartment studies provide quantitative evidence that chemical and genetic signals can be distributed differently among water, particles, and sediment. In one water–particle–sediment comparison, the reported total antibiotic concentrations were 1405.45 ng/L in surface water, 892.59 ng/g in suspended particulate matter, and 542.64 ng/g in sediment; sediment was nevertheless identified as the predominant ARG reservoir [60]. Around a tidal sewage outlet, ARG abundances ranged from 10^1^–10^5^ copies/mL in water and 10^5^–10^8^ copies/g in sediment, while maximum antibiotic concentrations reached 303 ng/L and 19,212 ng/kg, respectively; antibiotic–ARG correlations were strong in water but insignificant in sediment [68]. Aquaculture ponds likewise showed significant antibiotic–ARG correlations in sediment but not in source water or pond water [40]. These quantitative and statistical contrasts support compartment-dependent associations and are consistent with the redistribution of chemical and genetic signals among water, suspended particles, and sediment, a pattern relevant to the concept of risk displacement. This compartmental perspective is especially relevant in aquaculture and other managed waters, where exposure can involve water, particles, sediment, and biofilms concurrently.

### 4.3. What Field Mismatch Reveals and How to Test Post-Decline Legacy Persistence

The paired field evidence shows that chemical–genetic mismatch recurs across seasons, distances, and compartments. Direct tests of post-decline legacy persistence require a documented decline in source or selection pressure, sufficient follow-up, and concurrent measurement of continued loading, hydrology, biomass, and fecal inputs. Most existing studies were designed to characterize occurrence, association, or environmental gradients rather than post-decline recovery. They therefore identify the response patterns and environmental settings that should anchor source-reduction and before–after designs.

Seasonal reversals, downstream detectability, and cross-compartment divergence identify settings in which water-column parent-antibiotic concentrations are incomplete indicators of resistance-related genetic endpoints. These patterns inform a monitoring strategy: chemical and genetic trajectories should be paired with source indicators and hydrological context to distinguish continuing inputs, redistribution, and delayed genetic responses.

### 4.4. Mechanistic Evidence Explains Candidate Pathways for Chemical–Genetic Mismatch

Controlled experiments and focused mechanistic studies identify processes that can delay genetic recovery. ARG responses can remain dynamic after imposed antibiotic removal [77]. Extracellular DNA can persist in sediment and remain available for natural transformation [48,78]. Biofilm retention, host-community succession, sediment burial, and HGT can maintain genetic material after water-column parent compounds attenuate [77,79,80]. Hydrological retention and resuspension can also store and remobilize ARG-bearing particles [34,39,81]. Metals, disinfectants, and other stressors may maintain co-selection [24,25,82].

The mechanistic literature helps interpret mismatch. The following sections examine hydrodynamic memory, sediment and particulate reservoirs, transformation products, and multi-stressor co-selection. These interacting pathways can generate distinct chemical and genetic trajectories. Their relative importance can be resolved through source-aware, time-resolved field designs.

## 5. Interacting Mechanisms Generating and Sustaining Chemical–Genetic Mismatch

**Figure 3:**
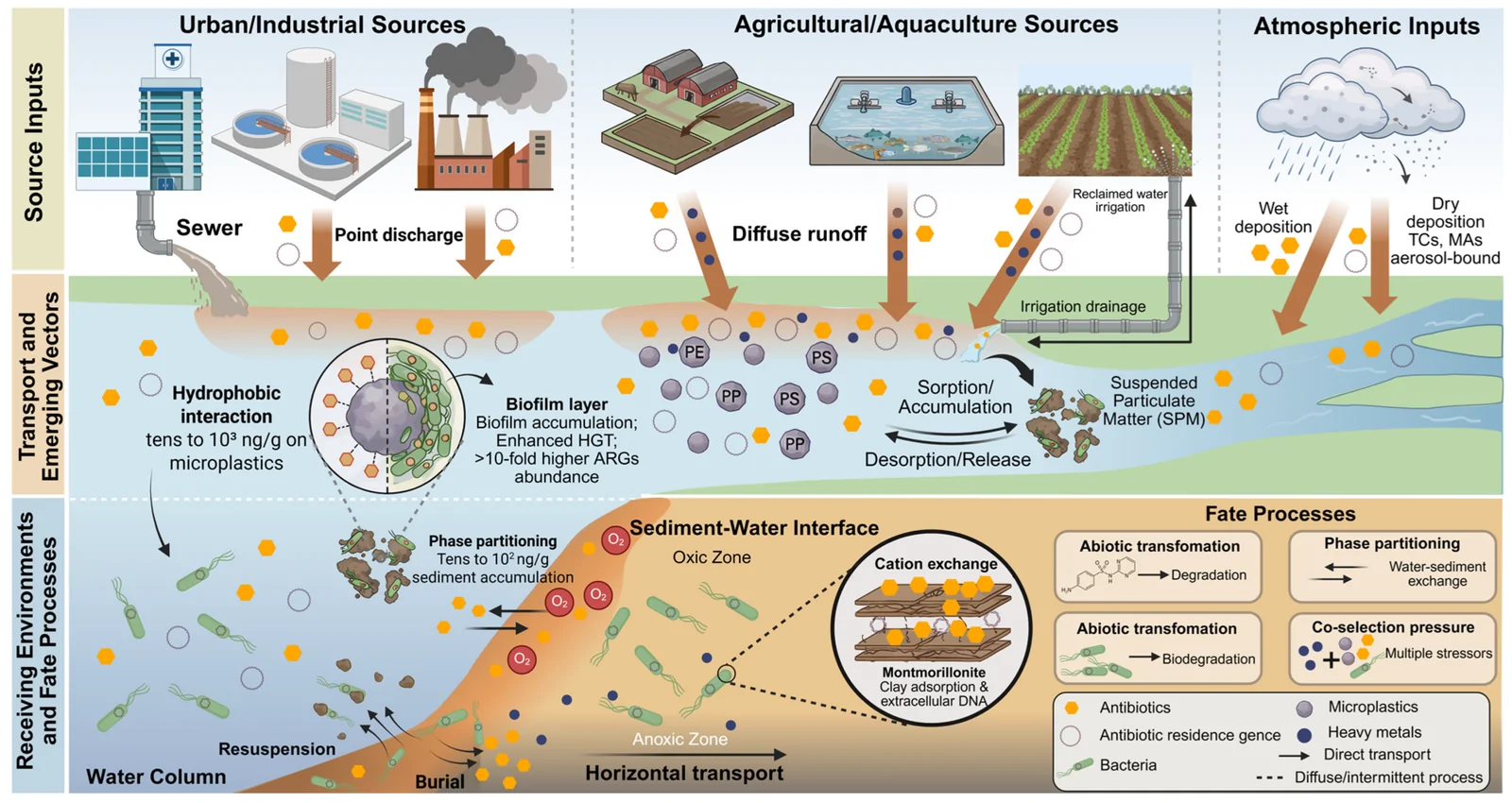
Sources, transport pathways, and environmental fate of antibiotics and antibiotic resistance genes (ARGs) in aquatic systems. The schematic contains three interconnected zones. The first covers source inputs: urban/industrial, agricultural/aquaculture, and atmospheric. The second covers transport and emerging vectors: microplastics, reclaimed water irrigation, and heavy metals. The third covers receiving environments and fate processes, including abiotic transformation, phase partitioning, biotic interactions, and co-selection pressure. The sediment-water interface shows redox stratification from oxic to anoxic conditions (Created in BioRender. Huang, L. (2026) https://BioRender.com/rsh7dnm). Representative quantitative evidence for selected processes illustrated here is summarized in Supplementary Table S3.

The interconnected source, transport, and fate processes that can generate chemical–genetic mismatch are summarized in Figure 3.

### 5.1. Hydrodynamic Redistribution and System Memory

Hydrodynamics can generate chemical–genetic mismatch through hydrological memory. Material from earlier inputs can be stored in particles, sediments, biofilms, and upstream compartments before later remobilization [36,39,81]. Residence time, advection, stratification, tidal exchange, and resuspension affect parent antibiotics and resistance-related genetic endpoints through different transport and retention pathways [34,35,83]. Paired monitoring should therefore resolve source intensity alongside chemical and genetic measurements.

The Three Gorges Reservoir Area illustrates how hydrodynamic models can guide interpretation. Model results indicate that source inputs, downstream transport, degradation, and sediment interaction redistribute antibiotic mass among locations and phases [84]. The case illustrates why phase-specific and source-resolved measurements are needed to link transport and retention to genetic endpoints.

In rivers, point-source inputs, downstream advection, tributary mixing, and dilution can produce steep local gradients in parent-antibiotic concentrations [85,86,87,88,89,90]. ARG measurements may show a similar spatial pattern near active discharges [55,61,62]. Farther downstream, chemical and genetic patterns may diverge as resistant organisms, particle-associated genes, and dissolved compounds move or decay at different rates [63,72]. Matched chemical and genetic sampling along the gradient may help distinguish transport effects from differences in source inputs.

Lakes and reservoirs have longer residence times, stratification, and repeated sediment–water exchange [91,92,93,94]. These processes can retain material from past inputs and delay recovery [49,95,96]. Estuaries also experience tidal mixing, salinity gradients, and particle trapping [34,40]. Across these systems, matched chemical and genetic trajectories are more informative for assessing differential attenuation, retention, and remobilization than isolated concentrations from different matrices. Salinity should also be treated as an ecological covariate rather than only as a hydrodynamic descriptor. Estuarine studies show that salinity gradients can restructure microbial communities, whereas their association with ARG profiles is system-dependent and may be weaker than the influence of anthropogenic inputs or mobile genetic elements [97,98].

Seasonal responses vary among systems. Dry-season enrichment has been reported in some settings [64,65,88]. Others show wet-season maxima, compound-specific responses, or gene-specific reversals [53,56,58,66,67,69,70]. Reduced dilution, temperature-dependent degradation, storm runoff, catchment loading, and shifts in bacterial biomass can act together [56,69,99]. Seasonal campaigns should therefore combine repeated sampling with hydrological and source indicators. Nutrient status can co-vary with seasonal runoff and wastewater loading and can modify microbial growth and the partitioning of resistance determinants. Experimental nutrient manipulations have produced contrasting responses between intracellular and extracellular ARG pools, indicating that nutrient enrichment can alter the biological context of a chemical–genetic trajectory without producing a uniform ARG response [100].

Storms, floods, droughts, reservoir drawdown, and tidal resuspension can alter both exposure and sampling context. They can mobilize particle-associated antibiotics and ARG-bearing material, dilute dissolved compounds, or reconnect stored and contemporary inputs [36,37,41,99]. Event-resolved mass balance and source indicators can reveal how these processes contribute to hydrological memory and chemical–genetic mismatch. Such event-driven redistribution can therefore change the timing and compartment of exposure even when routine water-column measurements suggest attenuation. Representative quantitative evidence for these event-driven storage and remobilization processes is summarized in Table 4.

### 5.2. Sediment, Suspended-Particle, and Biofilm Reservoirs

Sediments frequently contain substantial ARG inventories and may function as genetic reservoirs [44,101,102]. Their role is best evaluated using within-study normalized abundance, mass distribution, particle association, or experimentally resolved retention. These measures preserve the distinct denominators of water and sediment measurements.

Particle association and resuspension can displace chemical and genetic risk among sediment, particles, and water [39,81,103]. Storms can dilute dissolved signals while mobilizing catchment- and sediment-associated material [26,36,104]. The resulting pulses connect hydrology to resistance-related endpoints. Measurements of growth, transfer, and selection can then distinguish redistribution from in situ amplification.

Vertical ARG profiles vary with depositional history, sediment age, microbial biomass, redox conditions, and physical disturbance [49,50,96]. Surface enrichment, subsurface maxima, monotonic decline, and non-monotonic profiles can reflect different combinations of loading, burial, degradation, and resuspension [49,50,96]. Interpretation benefits from a common matrix and normalization scheme. At sediment–water interfaces, oxygen availability, redox state, organic matter, and nutrient loading are especially relevant because they regulate microbial metabolism, transformation conditions, and particle-associated retention. Nitrogen-driven eutrophication experiments further show that extracellular and intracellular ARG fractions can respond differently across water and sediment, with nutrient and co-occurring geochemical conditions contributing to the observed distribution patterns [105].

ARG distributions vary among aquatic-system types [49,50,51,106]. Lentic systems can retain ARG-bearing material, rivers can redistribute it longitudinally, and estuaries can trap or repeatedly resuspend particles [34,36,83]. Biofilms and extracellular DNA add further storage and transfer pathways [48,78,79,106]. These mechanisms can produce distinct chemical and genetic trajectories across compartments.

Bacteriophages provide an additional biological route for ARG persistence and dissemination. Phage-mediated transduction can move resistance determinants between bacterial hosts, while phage particles may protect packaged DNA from degradation and support transport across water, sediment, and biofilm compartments [107,108]. Field studies have detected ARGs in phage fractions of wastewater-impacted rivers, supporting their inclusion alongside plasmids and extracellular DNA in multi-compartment surveillance [62,109]. However, ARG detection in viral fractions does not by itself demonstrate functional transfer, and virome-based estimates can be inflated by bacterial DNA contamination or sequence misclassification [110]. Phage-associated resistance should therefore be interpreted together with host linkage, viral genomic context, and evidence of transfer activity.

Parent antibiotics partition among dissolved water, suspended particles, sediments, biofilms, and organisms according to compound properties and site chemistry [92,111,112]. Strong sorption by some fluoroquinolones and tetracyclines can support sediment retention [38,113]. Within-study mass distributions, partition coefficients, desorption behavior, and event-resolved release provide complementary evidence for this process [81,103,114].

Storms, floods, reservoir drawdown, overturn, and tidal currents can resuspend stored material and transiently increase water-column exposure [81,104,114]. Such pulses can redistribute chemical and genetic risk among compartments [39,68,81]. Hydrological state should therefore be recorded as part of chemical–genetic monitoring.

**Figure 4:**
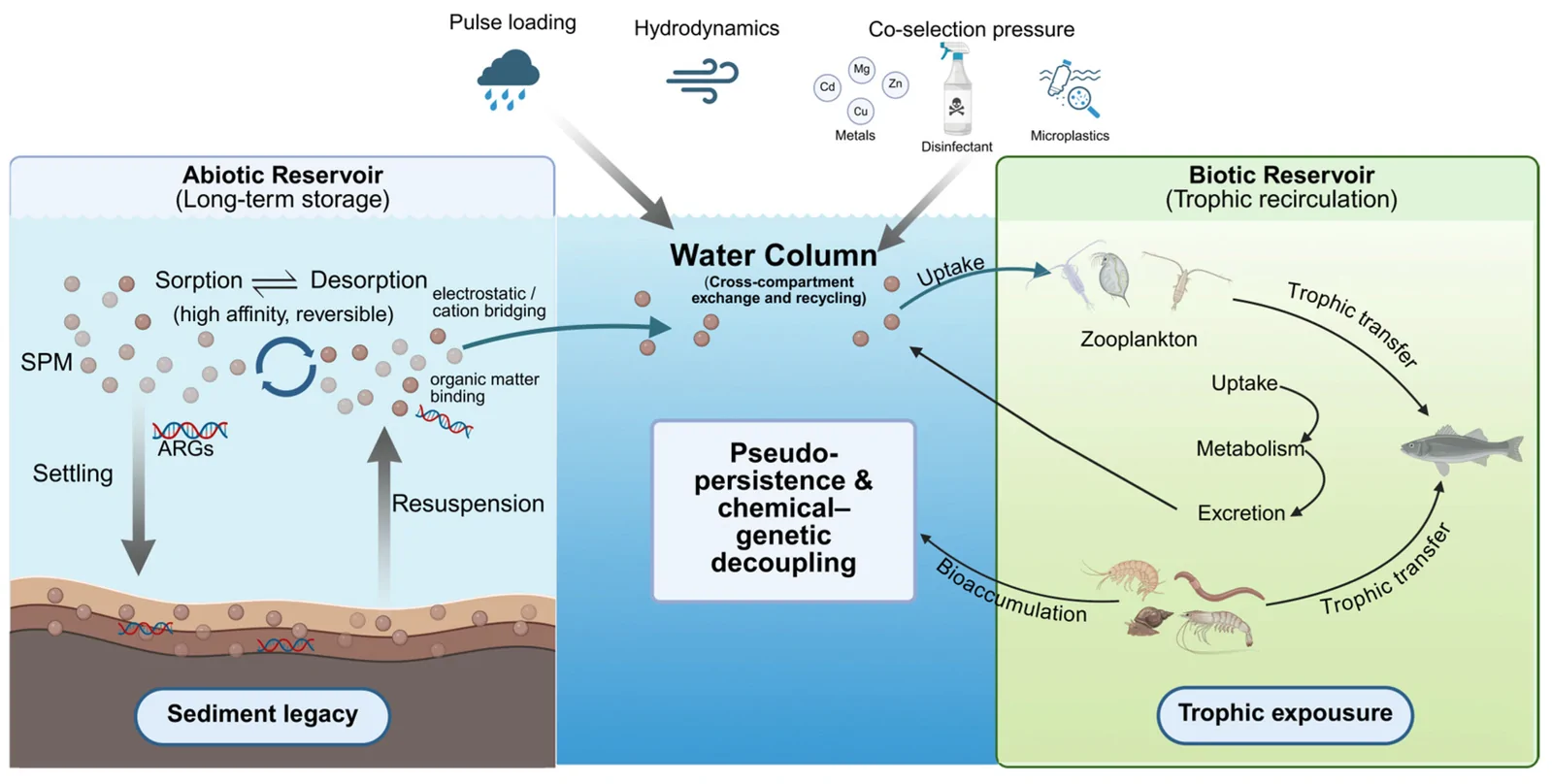
Conceptual dual-reservoir framework for chemical–genetic mismatch. Abiotic reservoirs include sediments and suspended particulate matter; biotic reservoirs include biofilms and pelagic or benthic organisms. Arrows represent candidate exchange pathways mediated by sorption–desorption, biological uptake–excretion, and hydrodynamic transport (Created in BioRender. Huang, L. (2026) https://BioRender.com/u2efw01).

Abiotic reservoirs, including sediments and SPM, can integrate exposure over longer periods than a single water sample [81,115,116]. Biofilms and other biotic compartments may do the same [79,117]. Exchange with the water column makes present-day concentrations an incomplete record of cumulative exposure [38,81,114]. It also offers a testable explanation for chemical–genetic mismatch. These storage and exchange pathways are synthesized in the dual-reservoir framework shown in Figure 4. Accordingly, exposure assessment for aquatic organisms should not rely on water samples alone when sediment, particles, or biofilms are connected to the local transport network. Representative quantitative evidence for storage and enrichment across abiotic and biotic reservoirs is summarized in Table 5.

### 5.3. Transformation Products and Residual Chemical Pressure

A decline in a measured parent antibiotic can coincide with continuing chemical pressure from transformation products, altered bioavailability, or partitioning into particles and sediments [119,120,121]. Some antimicrobial transformation products retain, and in individual cases may exceed, the parent compound’s ecotoxicological or selection-related activity [121,122,123]. Where these factors retain activity, they may contribute to threshold lowering. Chemical–genetic mismatch therefore warrants characterization of active products alongside parent compounds and genetic endpoints.

Transformation rates depend on antibiotic structure, pH, light, dissolved organic matter, turbidity, oxygen availability, and microbial-community composition [119,124,125,126]. Site-specific measurement of parent compounds, plausible transformation products, and associated co-selectors can connect chemical fate to genetic response. These physicochemical controls should not be treated as ancillary water-quality descriptors. Salinity and organic matter can influence sulfamethoxazole adsorption to suspended particles [103], while water-matrix composition can influence photolysis of selected antibiotic transformation products [127].

Laboratory and mesocosm studies point to extracellular DNA, particle-associated material, and ARG-carrying hosts as potential reservoirs that can complicate the interpretation of chemical attenuation [39,48,79]. These studies identify targets for field monitoring but do not by themselves establish field-supported legacy persistence.

Microbial transformation can couple chemical removal to shifts in community composition and MGEs [117,126,128,129]. Matched measurements of parent compounds, transformation products, hosts, mobile elements, and transfer or expression activity can test how this pathway contributes to chemical–genetic mismatch.

### 5.4. Multi-Stressor Co-Selection

**Figure 5:**
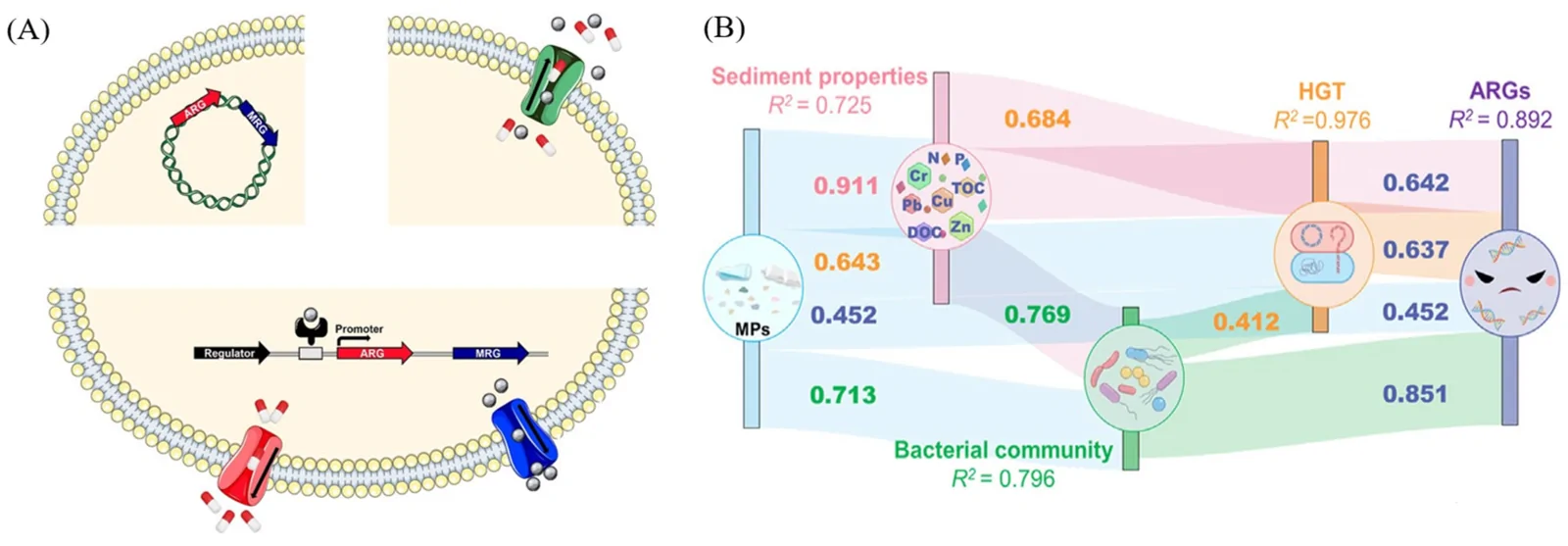
Candidate mechanisms of multi-stressor co-selection relevant to chemical–genetic mismatch. (**A**) Three heavy-metal co-selection mechanisms [82]: co-resistance, physical linkage of an antibiotic resistance gene (ARG) and a metal resistance gene (MRG) on the same mobile genetic element; cross-resistance, a single mechanism (illustrated by an efflux pump) conferring resistance to both antibiotics and metals; and co-regulation, shared control of ARG and MRG expression. Gray circles indicate metal ions and red-and-white symbols indicate antibiotics. (**B**) Structural-equation model linking microplastics (MPs), sediment properties, bacterial community, horizontal gene transfer (HGT), and ARG abundance in mariculture sediments [22]. Colored arrows represent modeled pathways; numerical values are standardized path coefficients, and R^2^ values indicate variance explained. The model represents direct and indirect MP effects on ARG abundance through sediment properties, bacterial communities, and HGT. Copyright 2025 Elsevier.

Heavy metals, including copper, zinc, and cadmium, frequently co-occur with antibiotics in aquatic systems [24,82,130]. Field observations and controlled studies report associations or experimentally induced increases in ARGs and intI1 under metal exposure [130,131,132]. Co-resistance, cross-resistance, and co-regulation can allow metal exposure to maintain or enrich resistance determinants independently of the parent antibiotic (Figure 5A) [82]. Such pathways may lower the effective parent-antibiotic concentration at which selection occurs [82,133]. Source-resolved, time-resolved field measurements are needed to establish their contribution in specific systems.

Microplastics can provide surfaces where bacteria, antibiotics, metals, and extracellular DNA co-occur [22,120,134,135]. A field structural-equation model associated microplastics with resistome propagation, primarily through host-community enrichment (Figure 5B) [22]. Laboratory evidence further indicates that weathering can alter sorption [120,136,137]. Microplastic-derived stress can also affect membrane permeability or genes associated with horizontal transfer [134,136,137,138]. These findings point to particle characteristics, host communities, and co-occurring stressors as linked measurements for evaluating plastisphere-associated resistance dynamics.

**Figure 6:**
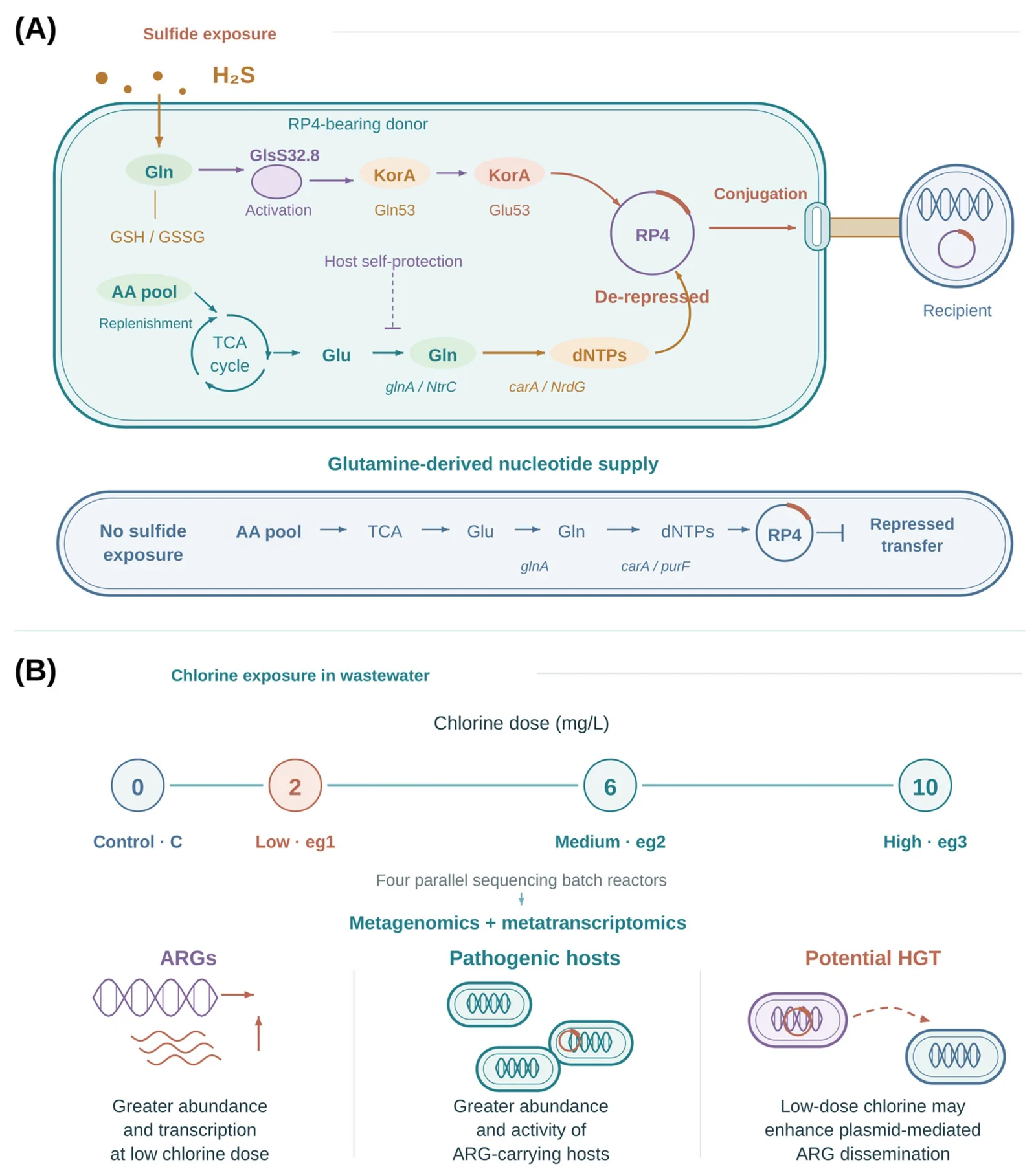
Controlled-study mechanisms by which chemical stressors may promote horizontal gene transfer (HGT). (**A**) Hydrogen-sulfide signaling and plasmid conjugation [23]. (**B**) Low-dose chlorine, oxidative stress, and HGT [25]. These mechanisms identify measurable pathways for field studies of chemical-genetic mismatch.

A controlled wastewater-system study showed that hydrogen sulfide can modify bacterial redox signaling and enhance plasmid conjugation (Figure 6A) [23]. Whether the same mechanism contributes materially in sedimentary or eutrophic anoxic zones requires field testing.

Sublethal disinfectant exposure provides another potential co-selection route. Controlled studies show that low-dose chlorine can induce oxidative and DNA-repair responses, alter host composition, and increase ARG abundance and transcriptional activity under tested treatment conditions (Figure 6B) [25]. This evidence supports joint monitoring of disinfectants, host dynamics, and mobile elements along treatment and receiving-water gradients.

The stressors described above can act through distinct pathways, including genetic linkage, shared efflux, host enrichment, altered conjugation, and stress responses [23,80,135,139]. When they co-occur, their combined effects may produce chemical–genetic trajectories that differ from those predicted by a single parent antibiotic.

These mechanisms provide routes through which ARGs may persist, diversify, or spread as parent-antibiotic concentrations decline [48,77,80,128]. Their relative contributions will vary with residence time, compartment structure, hydrodynamic disturbance, source continuity, and stressor profile. These system characteristics can guide targeted monitoring. In aquaculture settings, these co-occurring stressors can also complicate attribution of resistance responses to antibiotic exposure alone.

Supplementary Table S4 summarizes these system characteristics as testable expectations for targeted monitoring. It links hydrodynamic setting and stressor profile to candidate mismatch mechanisms and to the measurements most useful for resolving them. Supplementary Table S5 compiles study-reported non-antibiotic stressor conditions associated with resistance-related responses.

### 5.5. Mechanism Interactions and Emergent System Properties

These mechanisms can interact rather than operate independently. Hydrodynamic retention changes the duration and compartment of chemical exposure [34,35,83]. Particle and biofilm reservoirs alter host density and contact [22,79,80,134]. Transformation changes the identity of chemical pressure [119,121,123]. Co-selectors can sustain selection when a measured parent compound declines [24,25,82,135]. Their joint action can appear as chemical–genetic hysteresis, hydrological memory, risk displacement, or threshold lowering. These system properties are testable interpretations rather than retrospective labels.

Each system property specifies a different measurement strategy. Testing chemical–genetic hysteresis requires time-resolved paired trajectories after a documented chemical decline. Testing hydrological memory requires mass balance, storage, and remobilization measurements. Testing risk displacement requires linked measurements across water, particles, sediment, biofilm, and hosts. Testing threshold lowering requires co-selector and mixture measurements alongside parent antibiotics. These designs can discriminate among competing explanations without assigning a mechanism from co-occurrence alone. This process-based view helps explain environmental mismatch. It also highlights implications for aquatic-animal risk and management.

## 6. Risk Implications of Chemical–Genetic Mismatch for Aquatic Animals and One Health

### 6.1. Why Chemical Attenuation Is an Incomplete Risk Indicator

Parent-antibiotic concentrations remain relevant to toxicological exposure [17,18,140,141,142,143,144,145,146,147,148,149]. However, they do not capture the full resistance-related risk. Chemical attenuation can coincide with continued ARG loading, particle or sediment retention, mobile genetic elements, altered host communities, or transformation products [22,48,127]. Risk interpretation is strengthened by integrating chemical concentration, genetic abundance and mobility, host context, compartment, and source continuity. Declining water-column residues may therefore not coincide with reduced exposure to ARG-bearing particles, biofilms, or viable hosts. Risk assessment should distinguish chemical attenuation from changes in exposure and dissemination potential.

### 6.2. Aquatic Animal Exposure, Mobility, and Host Context

Aquatic organisms are both ecological receptors and potential links between environmental residues, resistance determinants, and human exposure [11,15,16,150]. Residue accumulation depends on compound and species [12,13,14]. Genetic risk depends more directly on viable hosts, mobile elements, expression, and transfer potential [15,16,150]. Integrating genomic linkage, host attribution, and persistence through processing and consumption will refine assessment of these exposure pathways. Exposure should therefore be considered across water, sediment, diet, and animal-associated microbial communities rather than inferred from water concentrations alone. Residue accumulation and genetic dissemination need not respond in parallel.

### 6.3. Aquaculture and the Boundaries of One Health Inference

Environmental, animal, food, and clinical resistomes are connected [151,152]. Clinically relevant genes and plasmids can move across these domains [151,152,153]. Genomic context, host resolution, source tracking, and temporal or epidemiological linkage can clarify transmission routes and directionality [29,151,153]. These measurements can support more specific One Health inference without treating environmental co-occurrence as a complete transmission map. In aquaculture systems, environmental ARG enrichment does not by itself demonstrate transmission into cultured animals or onward through the food chain. Stronger inference requires linkage among environmental sources, animal-associated hosts or mobile elements, and temporally resolved exposure or transfer evidence.

Accordingly, AMR assessment in aquatic production systems can pair environmental chemistry and resistome measurements with information on animal-associated microbial communities, viable resistant bacteria, mobile elements, and relevant exposure pathways. This combined approach links chemical–genetic mismatch to risk interpretation and aquaculture management.

## 7. Monitoring and Intervention Priorities for Aquatic AMR Management

The evidence synthesis translates into an operational agenda for aquatic resistance monitoring. Paired field studies repeatedly report seasonal, spatial, and compartmental mismatch. Parent-antibiotic concentrations should therefore be interpreted with source, hydrological, and genetic context. Monitoring can be organized around the four system properties introduced above. Priority designs are source-reduction or before–after studies. They should resolve continued loading and hydrological redistribution, normalize genetic endpoints to bacterial biomass or cell abundance, and measure MGEs, host context, transformation products, and major co-selectors. For aquaculture and aquatic-animal surveillance, this framework links environmental measurements to exposure pathways relevant to animals rather than treating water-column concentration as the sole management endpoint.

### 7.1. Expanding Monitoring Beyond Water-Phase Measurements

Although antibiotics and ARGs have been widely documented in aquatic systems, monitoring remains predominantly water-centric and spatiotemporally inconsistent [19,43]. Sediments and biofilms can retain resistance-related genetic material and may act as reservoirs [76,79,154]. Other compartments, including suspended particles, microplastics, hyporheic zones, and biota, should be included where they are connected to the local transport network.

Future monitoring campaigns should co-measure dissolved and particulate phases, representative biofilms and biota, and mobility markers such as integrons and other co-selective elements [28,155]. Sampling plans should include hydrological events, including floods, droughts, and typhoons, that change mixing and stratification [36,41,156]. Expanding beyond indicator genes to plasmid- and phage-associated vectors can better align surveillance with dissemination potential [62,157,158]. Where aquatic animals form part of the exposure pathway, host-associated measurements can be paired with environmental sampling to determine whether changes in surrounding water, particles, sediment, or biofilms are reflected in animal-associated resistance signals.

Future studies should, where feasible, combine genetic methods with conventional microbiological assays. Genetic analyses can identify resistance determinants and their genomic context, while culture-based isolation and antimicrobial susceptibility testing assess phenotypic resistance in viable microorganisms. Together, these approaches can help interpret genetic detections in light of microbial viability and phenotypic resistance.

Phage-associated ARGs should be interpreted with host linkage, viral genomic context, and transfer activity to distinguish carriage from demonstrated mobility [62,107,108,109,110].

Accordingly, paired monitoring should record the physicochemical and ecological covariates most relevant to each system. Temperature and pH should be measured routinely; dissolved oxygen or redox potential and organic matter are particularly important for sediment- and biofilm-linked settings; salinity should be resolved across estuarine gradients; and nutrient status should be characterized where eutrophication or wastewater inputs are plausible drivers [97,98,100,105]. These variables are best used as explanatory covariates alongside source, hydrology, microbial-community, and ARG/MGE measurements rather than interpreted as independent causal proxies.

### 7.2. Bridging Laboratory Models and Field Realities

Transformation pathways and metabolite risks remain poorly resolved under field conditions [4,121,127,159]. Photolysis, hydrolysis, and biodegradation are widely discussed [119,125,160,161]. Yet only a fraction of transformation products has been structurally identified and evaluated for ecological or antimicrobial-resistance-related risks [4,159]. Field conditions, including dissolved organic carbon, turbidity, metal oxides, microplastics, and sulfide-rich microenvironments, can alter reaction pathways [23,120,122,124]. They are therefore targets for combined chemical and genetic studies [135,162].

Minimum selective concentrations (MSCs) provide another route for connecting chemical exposure to resistance dynamics. Existing values derive largely from single-species and single-antibiotic experiments [163]. Metal co-selection, disinfectant pressure, interspecies competition, and biofilm structure may alter effective selection thresholds [82,133]. Suspect and non-target screening, redox-aware experiments, and field sampling during source changes and resuspension events can define these conditions more directly [164,165]. Experiments intended to inform aquaculture management should therefore reproduce environmentally realistic exposure histories and, where appropriate, include sediment, biofilm, or host-associated conditions that are absent from simplified single-compound systems.

### 7.3. Integrating Resistance Endpoints into Animal-Health and Environmental Risk Assessment

Conventional ecological risk assessment based on predicted environmental concentration/predicted no-effect concentration (PEC/PNEC) ratios primarily addresses apical toxicological effects [141,166,167]. Integrating resistance selection, HGT, and ARG mobility could extend this framework to resistance-related endpoints. MSCs and resistance-related PNECs can complement conventional ecotoxicological thresholds when their distinct purposes are retained [166,167].

A One Health-oriented exposure assessment can integrate trophic-transfer kinetics, perturbation of fish gut microbiomes, and food-borne mobility potential across the environmental-to-human transmission chain [168,169,170]. High-resolution approaches, including suspect screening, non-target liquid chromatography–high-resolution mass spectrometry (LC-HRMS), isotopic fingerprinting, and plasmid profiling, can better distinguish wastewater, livestock, aquaculture, septic, and atmospheric sources [171,172,173,174].

These priorities call for a shift from static concentration thresholds to time- and compartment-resolved risk assessment. They also move monitoring from single-compartment measurements to linked exposure pathways, and from toxicological endpoints alone to system-level interpretation that includes resistance selection, mobility, and host context.

### 7.4. Mechanism-Informed Intervention Options

Interventions should target the process identified by the monitoring design. Where continued loading dominates, priorities include antibiotic-use stewardship, source reduction, upgraded wastewater treatment, and management of aquaculture or livestock discharges. Where hydrological memory or risk displacement dominates, interventions should address storage, resuspension, and transport rather than water-column concentrations alone. Constructed wetlands, adsorption-based polishing, and other treatment trains can be evaluated jointly for parent antibiotics, ARGs, mobile elements, viable hosts, and transformation products [175,176,177,178].

Where source-resolved assessment identifies a sediment or particle reservoir, several strategies may be appropriate. These include monitored natural recovery, targeted removal, thin-layer capping, and reactive amendments. Their suitability depends on resuspension risk, habitat disturbance, and the fate of retained genetic material [179,180]. In catchments dominated by particle transport, riparian buffers, constructed wetlands, and particle-retention measures may reduce delivery during runoff events [175,176,178,181]. Before–after monitoring should determine whether interventions reduce loading, mobility, and host-associated genetic risk alongside water-column parent-antibiotic concentrations. In aquaculture systems, intervention design should connect antimicrobial stewardship and discharge control with monitoring of environmental reservoirs and host-associated endpoints.

### 7.5. Economic and Implementation Considerations

Cost-effectiveness cannot be inferred from chemical removal alone. A comparison should combine intervention cost with the mass of antibiotic and resistance-related risk avoided. It should also consider benefit duration, energy and material demand, land or habitat footprint, residuals, and monitoring burden. Interventions should be compared with a no-action or source-reduction baseline at the scale of the relevant compartment.

Source reduction and treatment upgrades are the first options when continued loading is documented. For diffuse runoff, riparian buffers, constructed wetlands, and particle-retention measures can be assessed where land and hydraulic capacity are available [176,178,181]. Where a sediment reservoir dominates, natural recovery, capping, reactive amendments, and targeted removal should be compared [179,180]. The comparison should consider resuspension risk, habitat disturbance, disposal requirements, and expected reductions in mobile or host-associated genetic endpoints. Reporting capital, operating, energy, land, and monitoring costs alongside paired chemical–genetic outcomes would enable future cross-system quantitative synthesis. For aquaculture implementation, feasibility should also consider production continuity, animal welfare, and the operational burden placed on farms or hatcheries.

## 8. Summary and Outlook

The most important observation from this review is that parent-antibiotic concentrations and resistance-related genetic endpoints do not follow a single, generalizable trajectory across aquatic systems. Among the 25 paired field studies, 16 provided Moderate and 9 Suggestive evidence for coupled, mismatched, or compartment-dependent responses, whereas none satisfied the stricter criterion for field-supported legacy persistence. Recurrent observations included seasonal reversals between antibiotics and ARGs, continued downstream detection of ARGs after chemical attenuation, and divergence among water, suspended particles, sediments, and biofilms. These patterns show that water-column parent-antibiotic concentrations alone are insufficient to characterize resistance-related genetic risk and that apparent persistence must be distinguished from continued loading, hydrological redistribution, and cross-compartment storage.

Three groups of mechanisms are especially important for interpreting these observations. First, storage and redistribution processes, including sediment and suspended-particle retention, biofilm accumulation, and hydrodynamic remobilization, can separate current water-column concentrations from earlier or compartment-specific exposure. Second, biological persistence and mobility, including extracellular DNA, viable ARG-bearing hosts, and horizontal gene transfer, can maintain or redistribute genetic determinants without closely tracking changes in dissolved parent-antibiotic concentrations. Third, residual or alternative selection pressures, including transformation products and non-antibiotic co-selectors such as metals and disinfectants, can sustain selection when the measured parent compound declines. These mechanisms may interact, but their relative contributions cannot yet be ranked reliably because most field studies lack source-resolved, time-resolved, and multi-compartment designs.

Management should therefore target the dominant source and process rather than ARG abundance alone. Where continued loading dominates, priorities are antimicrobial stewardship, reduction of wastewater, livestock, and aquaculture inputs, and treatment upgrades that are evaluated for both antibiotics and resistance-related endpoints. Where runoff and particle transport are important, riparian buffers, constructed wetlands, and particle-retention measures can reduce delivery to receiving waters. Where sediments act as important reservoirs and sources of remobilization, monitored natural recovery, capping, reactive amendments, or targeted removal may be considered according to resuspension risk and habitat impact. In aquaculture systems, antimicrobial-use reduction and discharge control should be coupled with monitoring of water, sediment, biofilms, viable hosts, ARGs, MGEs, and relevant co-selectors. Before–after and source-reduction studies are essential for determining whether these interventions reduce not only chemical exposure but also mobile and host-associated resistance signals. As climate change, urbanization, and expanding aquaculture continue to reshape aquatic ecosystems, treating aquatic animals as ecological receptors, vectors, and monitoring and management targets for antimicrobial resistance will be central to safeguarding aquatic animal health and food safety in this changing world.

## Figures and Tables

**Table 1:** Operational checklist used to interpret and grade paired antibiotic–ARG field evidence.

Assessment Domain	Moderate Evidence	Suggestive Evidence
Paired measurements	Chemical and ARG/MGE observations are directly aligned by site, period, event, intervention, or compartment.	Pairing remains informative but is partially constrained; insufficient pairing excludes a study from core matched-response coding.
Reference condition/comparator	A clear within-study seasonal, spatial, intervention, or compartmental comparator supports the response direction.	The comparator is weak, unbalanced, or incompletely resolved but still permits a provisional paired response.
Normalization	The genetic endpoint denominator or normalization supports within-study directional comparison.	The denominator choice or inconsistent normalization materially limits interpretation.
Matrix comparability	The same matrix is used, or a cross-compartment comparison is explicitly interpretable.	Cross-matrix comparison or non-comparable units/denominators materially constrains inference.
Temporal/spatial resolution	Sampling resolution is sufficient to determine the paired response direction.	Sparse, aggregated, or scale-dependent sampling leaves the response only partly resolved.
Source attribution/hydrological context	Remaining uncertainty about loading, mixing, or hydrology does not prevent interpretation of the observed response.	Source continuity, mixing, or hydrological ambiguity materially constrains interpretation of a single response pattern.

Note: Grades describe confidence in the interpretability of the observed paired response, not overall study quality, causal certainty, or evidence for legacy persistence. Minimum core eligibility required aligned paired measurements, a study-supported comparator or reference condition, and sufficient result-level information to interpret a joint chemical–genetic response without constructing a new contrast not supported by the original study design. Failure to meet these minimum requirements excluded a study from core matched-response coding.

**Table 2:** Summary of the 25 core field studies used for matched antibiotic–ARG/MGE response coding.

Study	System and Design	Paired Response and Observed Pattern	Evidence Grade
Garcia-Torné et al. (2025) [53]	Spanish Mediterranean wastewater-impacted waters; seasonal paired survey	Antibiotics peaked in winter; ARG levels were similar between seasons, but ARG detection diversity was greater in summer. Pattern: Seasonal mismatch.	Moderate
Zhang et al. (2024) [54]	Five Chinese rivers spanning rural–urban land use; multi-river comparison	Quinolones and ARG/MGE richness were greatest in more urbanized rivers. Pattern: Coupled spatial enrichment.	Suggestive
Beltrán de Heredia et al. (2023) [55]	Three WWTP-impacted river basins; upstream–downstream × flow-period survey	Antibiotic contamination and most ARGs increased below outfalls and were generally higher at low water. Pattern: Coupled spatial and seasonal enrichment.	Moderate
Gao et al. (2022) [56]	Two subtropical river basins, South China; wet–dry paired survey	Water antibiotics peaked in the dry season, whereas relative water ARG abundance peaked in the wet season; sediment differed. Pattern: Seasonal mismatch.	Moderate
Li et al. (2020) [57]	Shanghai river–reservoir drinking-water system; inflow-to-purification survey	Antibiotics, ARGs and MGEs declined through reservoir ecological purification. Pattern: Coupled attenuation through ecological purification.	Moderate
Yuan et al. (2019) [40]	Hangzhou Bay estuarine aquaculture ponds; water–sediment survey	Antibiotics varied among source water, ponds and sediment; ARG associations occurred in sediment but not water. Pattern: Compartment-dependent association.	Suggestive
Xu et al. (2018) [58]	Liaohe catchment; May–October water–sediment survey	Tetracyclines, sulfonamides, ARGs and resistant bacteria showed compound- and gene-specific seasonal patterns. Pattern: Mixed seasonal response.	Suggestive
Gao et al. (2026) [59]	Twenty-six Bohai Rim rivers; cross-media survey	Antibiotic and ARG distributions differed between water and sediment, although ARG values across the two matrices were reported using non-comparable denominators. Pattern: Compartmental mismatch with non-comparable units.	Suggestive
Ohore et al. (2024) [60]	Surface water, suspended particles and sediment; matched compartment survey	Antibiotic mass was greatest in surface water, but sediment was the predominant ARG reservoir. Pattern: Compartment-specific mismatch.	Moderate
Wang et al. (2022) [61]	Qinhuai River below a WWTP; near-discharge versus 2 km sediment survey	Sediment antibiotics and ARGs were both higher near the WWTP discharge than 2 km downstream. Pattern: Coupled spatial attenuation.	Moderate
Lekunberri et al. (2017) [62]	Ter River receiving treated wastewater; upstream–downstream survey	Antibiotics and several ARGs increased below the WWTP, including bacterial, phage and plasmid fractions. Pattern: Coupled spatial enrichment.	Moderate
Berglund et al. (2015) [63]	Stångån River receiving WWTP effluent; three-month monitoring	Low-ng/L antibiotics occurred in effluent/downstream water; several ARGs and intI1 increased in downstream sediment-associated material. Pattern: Spatial mismatch with cross-matrix limitation.	Suggestive
Liang et al. (2020) [64]	Poyang Lake surface waters; wet–dry spatial survey	Total antibiotics and ARG abundance were both higher in the dry season. Pattern: Coupled seasonal enrichment.	Moderate
Wu et al. (2022) [65]	Chishui River; dry–rainy spatial survey	Antibiotics and most ARGs peaked in the dry season, with gene-specific exceptions. Pattern: Coupled seasonal enrichment with gene-specific exceptions.	Moderate
Jiang et al. (2021) [66]	Agriculture-impacted rural river; multi-season water–sediment survey	Seasonal antibiotic patterns did not consistently track corresponding ARGs or MGEs. Pattern: Gene-specific seasonal mismatch.	Moderate
Zhang et al. (2024) [67]	Chinese river–estuary continuum; seasonal land-use/hydrodynamic gradient	Total antibiotics correlated with overall ARG pollution, but individual ARGs varied with season, land use and hydrodynamics. Pattern: Mixed spatial-seasonal association under hydrodynamic forcing.	Suggestive
Lou et al. (2026) [68]	Yangtze Estuary sewage outlet; tidal-cycle water–sediment survey	Antibiotic–ARG correlations were strong in water but absent in sediment and varied with tidal phase. Pattern: Tidal and compartment-specific response.	Suggestive
Yang et al. (2024) [69]	Macrolide-impacted urban river; three hydrological periods	Macrolides and MLS ARGs rose together along the dry-season gradient, but the ARG gradient reversed in the wet period. Pattern: Season-dependent response reversal.	Moderate
Chen et al. (2024) [70]	Songliao Basin; three hydrological periods including ice cover	Antibiotics peaked in the dry season; relative ARG abundance and resistant bacteria peaked in the wet season. Pattern: Seasonal mismatch.	Moderate
Kerrigan et al. (2018) [71]	Two rivers and a wastewater-impacted lake, USA; multiscale sediment survey	Antibiotic–ARG correlations occurred along a short lake transect but disappeared across larger river scales. Pattern: Scale-dependent loss of association.	Moderate
Sabri et al. (2020) [72]	Dutch WWTP-receiving river; one-year survey to 20 km downstream	Measured antibiotics declined or remained stable downstream; ARGs remained detectable to 20 km and sul1 declined little. Pattern: Spatial mismatch.	Moderate
Cho et al. (2023) [73]	US mixed-use watershed with three WWTPs; upstream–downstream survey	Neither antibiotics nor ARGs differed significantly between upstream and downstream waters. Pattern: No resolved upstream–downstream contrast.	Suggestive
Tsholo et al. (2025) [74]	South African WWTPs and receiving rivers; paired effluent–river survey	Antibiotic residues and ARGs varied heterogeneously by site, month and gene, without a consistent downstream trajectory. Pattern: Spatially heterogeneous response.	Suggestive
Milaković et al. (2019) [75]	Sava River below azithromycin manufacture; two-season sediment survey	Macrolides, corresponding ARGs and intI1 were enriched below the manufacturing discharge, in some cases to 11 km. Pattern: Coupled spatial enrichment.	Moderate
Luo et al. (2010) [76]	Haihe River; 72 km matched water–sediment survey	Normalized sul1 and sul2 abundance correlated positively with summed sulfonamides across the river. Pattern: Coupled cross-sectional association.	Moderate

Note: Grades indicate confidence in the interpretability of the observed paired chemical–genetic response, not overall study quality or causal certainty. ARG, antibiotic resistance gene; MGE, mobile genetic element; WWTP, wastewater treatment plant. Full study-specific design context and bibliographic details are provided in Supplementary Table S2.

**Table 3:** Representative field evidence for concordant antibiotic and ARG/MGE responses across environmental gradients.

Environmental Setting	Representative Study	Antibiotic Evidence	ARG/MGE Evidence
WWTP discharge	Wang et al. (2022) [61]	Sediment antibiotics: 37.86–76.11 μg/kg dw; significantly higher near the discharge	ARG abundance was also significantly higher near the discharge; 245 ARGs detected
Seasonal concentration	Liang et al. (2020) [64]	Total antibiotics were higher in the dry season; sulfonamides accounted for >50% of total antibiotics	Total ARG abundance was also higher in the dry season; 12 ARGs detected (19.2–100% detection frequency)
Urbanization gradient	Zhang et al. (2024) [54]	QNs: 16.39–85.98 → 23.34–1188 → 160.1–2151 ng/g (rural → rural–urban → urban)	ARG subtypes: 46–79 → 59–61 → 145–161; MGE subtypes: 29–35 → 29–30 → 55–60
Pharmaceutical discharge	Milaković et al. (2019) [75]	AZI: 4.60 → 9307 μg/kg (winter); <LOD → 23,685 μg/kg (summer), upstream → discharge	ARG relative abundance: ~10^−5^–10^−4^ → ~10^−2^–10^−1^ copies/rrn, upstream → discharge

**Table 4:** Representative quantitative evidence for hydrodynamically driven storage and remobilization.

Hydrodynamic Process	Study System/Condition	Representative Quantitative Evidence	Interpretation
Storm runoff	Urban inland stream; three storm runoff events	Five target ARGs were tracked across three storms. Between 37 and 121 different ARGs were detected per stream sample, and storm loads of all five monitored ARGs were significantly greater than during equivalent background periods [26].	Storm runoff can transiently increase downstream ARG loading and alter the resistome profile.
Extreme flooding	Chaohu Lake and surrounding submerged polders after the extreme 2020 Yangtze River Basin flood	Antibiotic concentrations ranged from 5.0 to 317 ng/L, and total antibiotic storage in submerged polders was estimated at 27.9 kg [36].	Flood storage can retain antibiotic mass and provide a source for subsequent remobilization during drainage.
Wind-wave resuspension	Simulated water-sediment system; suspended solids of 100, 500, and 1000 mg/L	Maximum sediment adsorption capacity reached 51.73 mg/kg, and NOR accumulation in surface sediment during resuspension was approximately 14-fold that of SMX [81].	Resuspension can strongly alter antibiotic partitioning and redistribution, with compound-specific effects.
Sediment remobilization	Small-stream sediment-water system; worst-case resuspension scenario	Sediment bacterial abundance exceeded water-column abundance by 1–2 log units. Resuspension of the upper 5 cm within 1 h yielded a modeled downstream/upstream suspended bacterial-load ratio of approximately 22 [39].	Sediment disturbance can markedly increase water-column loads of sediment-associated bacteria; the estimate is scenario-specific rather than a field-wide effect size.

Note: Values are study-specific examples and are not pooled effect estimates. ARG, antibiotic resistance gene; MGE, mobile genetic element; NOR, norfloxacin; SMX, sulfamethoxazole.

**Table 5:** Representative quantitative evidence for abiotic and biotic reservoirs relevant to chemical–genetic mismatch.

Reservoir/Process	Study System/Condition	Representative Quantitative Evidence	Relevance to the Dual-Reservoir Framework
Sediment/suspended particles	Simulated Lake Taihu water–sediment system under wind–wave resuspension	Suspended solids were 100, 500, and 1000 mg/L under background, moderate, and strong disturbance. Maximum sediment adsorption capacity reached 51.73 mg/kg, and NOR accumulation in surface sediment was approximately 14-fold that of SMX [81]	Demonstrates quantitatively that disturbance and sorption can concentrate some antibiotics in the abiotic reservoir.
Water–sediment–biofilm partitioning	Six urban lakes in Nanjing; 21 antibiotics across water, sediment, and biofilm	Quinolones occurred at 10.63–38.78 ng/L in water, 0.40–12.77 ng/g in sediment, and ND–73.30 ng/g in biofilms. Biofilm quinolone concentrations correlated with human-activity intensity (r = 0.92, *p* < 0.01) [114]	Shows differential chemical accumulation across connected water, sediment, and biofilm compartments.
Natural biofilm ARG reservoir	Weihe River; matched biofilm and sediment survey	sulI ranged from 7.50 × 10^5^ to 2.01 × 10^9^ copies/g in biofilms and from 1.46 × 10^5^ to 1.12 × 10^8^ copies/g in sediments. Mean abundances of the multidrug-resistance genes floR and cmlA were 8.76 × 10^8^ copies/g in biofilms and 7.34 × 10^7^ copies/g in sediments [118]	Provides direct quantitative evidence that natural biofilms can concentrate ARGs relative to adjacent sediment.

Note: Values are study-specific examples and are not pooled effect estimates. Concentration and abundance units differ among matrices and endpoints. ARG, antibiotic resistance gene; NOR, norfloxacin; SMX, sulfamethoxazole.

## Data Availability

The data supporting this review are included in the article and Supplementary Tables S1–S5.

## Supplementary Materials

The following supporting information can be downloaded at: https://www.mdpi.com/article/10.3390/ani16193070/s1. Supplementary Tables S1–S5 are provided as supporting information with this manuscript. References [182,183,184,185,186,187,188,189,190,191,192,193,194,195,196,197,198,199,200,201,202,203,204,205,206,207] are cited in the Supplementary Materials.
