# Supplementary material for: Beyond Co-Occurrence: A Framework for Interpreting Antibiotic–ARG Decoupling in Aquatic Systems and Its Implications for Aquatic Animal Health in a Changing World
Source: Animals (Basel). 2026 Sep 29;16(19):3070. doi: 10.3390/ani16193070 (PMC13663438; doi:10.3390/ani16193070)
Supplement: Supplementary file 1 [file animals-16-03070-s001.zip › animals-4559577-supplementary.pdf]

SUPPLEMENTARY INFORMATION

Table S1. Complete database search strings, retrieval dates, and retrieval counts.

Database	Search label	Search date	Retrieved records	Search string	Notes
Web of Science Core Collection	WOS_main_aquatic_search	2026-07-23	8395	TS=(("antibiotic resistance gene" OR "antibiotic resistance genes" OR "antimicrobial resistance gene" OR "antimicrobial resistance genes" OR resistome* OR "mobile genetic element" OR "mobile genetic elements" OR integron*) AND (antibiotic* OR antimicrobial*) AND (river* OR stream* OR lake* OR reservoir* OR estuar* OR "surface water" OR "surface waters" OR watershed* OR sediment* OR biofilm* OR "suspended particulate matter" OR aquatic)) NOT TS=(patient* OR "clinical trial" OR "clinical trials")	Topic-field syntax; timespan 1900–2026.
Scopus	Scopus_main_aquatic_search	2026-07-24	8235	TITLE-ABS-KEY(("antibiotic resistance gene" OR "antibiotic resistance genes" OR "antimicrobial resistance gene" OR "antimicrobial resistance genes" OR resistome* OR "mobile genetic element" OR "mobile genetic elements" OR integron*) AND (antibiotic* OR antimicrobial*) AND (river* OR stream* OR lake* OR reservoir* OR estuar* OR "surface water" OR "surface waters" OR watershed* OR sediment* OR biofilm* OR "suspended particulate matter" OR aquatic)) AND NOT TITLE-ABS-KEY(patient* OR "clinical trial" OR "clinical trials") AND PUBYEAR < 2027	Advanced document-search syntax; records through 2026.
PubMed	P1_paired_aquatic	2026-07-23	3823	(<br>("antibiotic resistance gene"[Title/Abstract] OR "antibiotic resistance genes"[Title/Abstract]<br>OR "antimicrobial resistance gene"[Title/Abstract] OR "antimicrobial resistance genes"[Title/Abstract]<br>OR resistome[Title/Abstract] OR "mobile genetic element"[Title/Abstract]<br>OR "mobile genetic elements"[Title/Abstract])<br>AND<br>(antibiotic[Title/Abstract] OR antibiotics[Title/Abstract] OR antimicrobial[Title/Abstract]<br>OR antimicrobials[Title/Abstract] OR "antibiotic residue"[Title/Abstract]<br>OR "antibiotic residues"[Title/Abstract])<br>AND<br>(river[Title/Abstract] OR rivers[Title/Abstract] OR stream[Title/Abstract]<br>OR streams[Title/Abstract] OR lake[Title/Abstract] OR lakes[Title/Abstract]<br>OR reservoir[Title/Abstract] OR reservoirs[Title/Abstract] OR estuary[Title/Abstract]<br>OR estuarine[Title/Abstract] OR "surface water"[Title/Abstract]<br>OR "surface waters"[Title/Abstract] OR aquatic[Title/Abstract])<br>) NOT (patient[Title/Abstract] OR patients[Title/Abstract] OR clinical[Title/Abstract])	Paired aquatic discovery.
PubMed	P2_decoupling_designs	2026-07-23	1007	(<br>("antibiotic resistance gene"[Title/Abstract] OR "antibiotic resistance genes"[Title/Abstract]<br>OR resistome[Title/Abstract] OR "mobile genetic element"[Title/Abstract]<br>OR "mobile genetic elements"[Title/Abstract])<br>AND<br>(antibiotic[Title/Abstract] OR antibiotics[Title/Abstract]<br>OR "antibiotic residue"[Title/Abstract] OR "antibiotic residues"[Title/Abstract])<br>AND<br>(seasonal[Title/Abstract] OR temporal[Title/Abstract] OR longitudinal[Title/Abstract]<br>OR gradient[Title/Abstract] OR downstream[Title/Abstract] OR upstream[Title/Abstract]<br>OR attenuation[Title/Abstract] OR persistence[Title/Abstract] OR sediment[Title/Abstract]<br>OR sediments[Title/Abstract] OR biofilm[Title/Abstract])<br>AND<br>(river[Title/Abstract] OR lake[Title/Abstract] OR reservoir[Title/Abstract]<br>OR estuary[Title/Abstract] OR estuarine[Title/Abstract] OR "surface water"[Title/Abstract]<br>OR aquatic[Title/Abstract] OR watershed[Title/Abstract])<br>) NOT (patient[Title/Abstract] OR patients[Title/Abstract] OR clinical[Title/Abstract])	Temporal, spatial, and cross-compartment designs.
PubMed	P3_mechanistic_support	2026-07-23	1631	(<br>("antibiotic resistance gene"[Title/Abstract] OR "antibiotic resistance genes"[Title/Abstract]<br>OR resistome[Title/Abstract] OR "horizontal gene transfer"[Title/Abstract])<br>AND<br>(antibiotic[Title/Abstract] OR antibiotics[Title/Abstract]<br>OR "selective concentration"[Title/Abstract] OR "sub-inhibitory"[Title/Abstract]<br>OR subinhibitory[Title/Abstract])	Mechanistic supporting evidence.

Database	Search label	Search date	Retrieved records	Search string	Notes
				AND<br>(persistence[Title/Abstract] OR selection[Title/Abstract] OR enrichment[Title/Abstract]<br>OR amplification[Title/Abstract] OR transfer[Title/Abstract] OR coselection[Title/Abstract]<br>OR "co-selection"[Title/Abstract])<br>AND<br>(aquatic[Title/Abstract] OR water[Title/Abstract] OR sediment[Title/Abstract]<br>OR biofilm[Title/Abstract] OR microcosm[Title/Abstract] OR mesocosm[Title/Abstract])<br>) NOT (patient[Title/Abstract] OR patients[Title/Abstract] OR clinical[Title/Abstract])	

Note: The screening process and DOI-first, normalized-title deduplication rule are reported in the Methods.

The three PubMed searches retrieved 6,461 records before within-database deduplication. After PMID-based deduplication across the three PubMed search sets, 4,696 unique PubMed records remained and were used in the cross-database total reported in the main text.

Table S2. Paired field evidence for antibiotic–ARG response patterns across aquatic systems.

Study (Ref.)	System and design	Paired antibiotic–ARG/MGE response	Key design context	Observed pattern	Grade
Garcia-Torné et al. (2025) [1]	Spanish Mediterranean wastewater-impacted waters; seasonal paired survey	Antibiotics peaked in winter; ARG levels were similar between seasons, but ARG detection diversity was greater in summer.	Season, temperature, sunlight and wastewater input co-varied.	Seasonal mismatch	M
Zhang et al. (2024) [2]	Five Chinese rivers spanning rural–urban land use; multi-river comparison	Quinolones and ARG/MGE richness were greatest in more urbanized rivers.	Cross-sectional urbanization comparison; sources and hydrology were incompletely resolved.	Coupled spatial enrichment	S
Beltrán de Heredia et al. (2023) [3]	Three WWTP-impacted river basins; upstream–downstream × flow-period survey	Antibiotic contamination and most ARGs increased below outfalls and were generally higher at low water.	Active effluent loading continued; flow conditions varied.	Coupled spatial and seasonal enrichment	M
Gao et al. (2022) [4]	Two subtropical river basins, South China; wet–dry paired survey	Water antibiotics peaked in the dry season, whereas relative water ARG abundance peaked in the wet season; sediment differed.	Wet/dry loading and hydrology changed together.	Seasonal mismatch	M
Li et al. (2020) [5]	Shanghai river–reservoir drinking-water system; inflow-to-purification survey	Antibiotics, ARGs and MGEs declined through reservoir ecological purification.	No source-cessation or post-treatment recovery interval.	Coupled attenuation through ecological purification	M
Yuan et al. (2019) [6]	Hangzhou Bay estuarine aquaculture ponds; water–sediment survey	Antibiotics varied among source water, ponds and sediment; ARG associations occurred in sediment but not water.	Pond age, stocking density and matrix differences confounded inference.	Compartment-dependent association	S
Xu et al. (2018) [7]	Liaohe catchment; May–October water–sediment survey	Tetracyclines, sulfonamides, ARGs and resistant bacteria showed compound- and gene-specific seasonal patterns.	Compound/gene specificity and hydrology limited a single trajectory.	Mixed seasonal response	S
Gao et al. (2026) [8]	Twenty-six Bohai Rim rivers; cross-media survey	Antibiotic and ARG distributions differed between water and sediment, although ARG values across the two matrices were reported using non-comparable denominators.	Water–sediment units and denominators were not directly comparable.	Compartmental mismatch with non-comparable units	S
Ohore et al. (2024) [9]	Surface water, suspended particles and sediment; matched compartment survey	Antibiotic mass was greatest in surface water, but sediment was the predominant ARG reservoir.	Matrix partitioning and microbial community differences were not source cessation.	Compartment-specific mismatch	M
Wang et al. (2022) [10]	Qinhuai River below a WWTP; near-discharge versus 2-km sediment survey	Sediment antibiotics and ARGs were both higher near the WWTP discharge than 2 km downstream.	The WWTP remained an active source.	Coupled spatial attenuation	M
Lekunberri et al. (2017) [11]	Ter River receiving treated wastewater; upstream–downstream survey	Antibiotics and several ARGs increased below the WWTP, including bacterial, phage and plasmid fractions.	Chronic treated-wastewater loading continued.	Coupled spatial enrichment	M
Berglund et al. (2015) [12]	Stångån River receiving WWTP effluent; three-month monitoring	Low-ng/L antibiotics occurred in effluent/downstream water; several ARGs and intI1 increased in downstream sediment-associated material.	Chemical water measurements were compared with sediment-associated genetics under continued discharge.	Spatial mismatch with cross-matrix limitation	S
Liang et al. (2020) [13]	Poyang Lake surface waters; wet–dry spatial survey	Total antibiotics and ARG abundance were both higher in the dry season.	Hydrological concentration and tributary inputs co-varied seasonally.	Coupled seasonal enrichment	M
Wu et al. (2022) [14]	Chishui River; dry–rainy spatial survey	Antibiotics and most ARGs peaked in the dry season, with gene-specific exceptions.	Hydrology, metals, MGEs and host communities co-varied.	Coupled seasonal enrichment with gene-specific exceptions	M

Study (Ref.)	System and design	Paired antibiotic–ARG/MGE response	Key design context	Observed pattern	Grade
Jiang et al. (2021) [15]	Agriculture-impacted rural river; multi-season water–sediment survey	Seasonal antibiotic patterns did not consistently track corresponding ARGs or MGEs.	Sediment transport, rural inputs and co-selectors remained plausible.	Gene-specific seasonal mismatch	M
Zhang et al. (2024) [16]	Chinese river–estuary continuum; seasonal land-use/hydrodynamic gradient	Total antibiotics correlated with overall ARG pollution, but individual ARGs varied with season, land use and hydrodynamics.	Hydrodynamics, metals, nutrients and communities co-varied.	Mixed spatial-seasonal association under hydrodynamic forcing	S
Lou et al. (2026) [17]	Yangtze Estuary sewage outlet; tidal-cycle water–sediment survey	Antibiotic–ARG correlations were strong in water but absent in sediment and varied with tidal phase.	Active sewage input, tides and resuspension continued.	Tidal and compartment-specific response	S
Yang et al. (2024) [18]	Macrolide-impacted urban river; three hydrological periods	Macrolides and MLS ARGs rose together along the dry-season gradient, but the ARG gradient reversed in the wet period.	Rainfall, nutrients, MGEs and bacterial composition changed concurrently.	Season-dependent response reversal	M
Chen et al. (2024) [19]	Songliao Basin; three hydrological periods including ice cover	Antibiotics peaked in the dry season; relative ARG abundance and resistant bacteria peaked in the wet season.	Biomass, temperature, hydrology and MGEs were alternative drivers.	Seasonal mismatch	M
Kerrigan et al. (2018) [20]	Two rivers and a wastewater-impacted lake, USA; multiscale sediment survey	Antibiotic–ARG correlations occurred along a short lake transect but disappeared across larger river scales.	Associations were scale dependent and cross-sectional.	Scale-dependent loss of association	M
Sabri et al. (2020) [21]	Dutch WWTP-receiving river; one-year survey to 20 km downstream	Measured antibiotics declined or remained stable downstream; ARGs remained detectable to 20 km and sul1 declined little.	Effluent input continued; antibiotics covered only part of ARG monitoring.	Spatial mismatch	M
Cho et al. (2023) [22]	US mixed-use watershed with three WWTPs; upstream–downstream survey	Neither antibiotics nor ARGs differed significantly between upstream and downstream waters.	Multiple sources, mixing and unbalanced comparisons limited inference.	No resolved upstream–downstream contrast	S
Tsholo et al. (2025) [23]	South African WWTPs and receiving rivers; paired effluent–river survey	Antibiotic residues and ARGs varied heterogeneously by site, month and gene, without a consistent downstream trajectory.	Runoff and non-WWTP sources were incompletely resolved.	Spatially heterogeneous response	S
Milaković et al. (2019) [24]	Sava River below azithromycin manufacture; two-season sediment survey	Macrolides, corresponding ARGs and intI1 were enriched below the manufacturing discharge, in some cases to 11 km.	Industrial discharge and co-selectors remained active.	Coupled spatial enrichment	M
Luo et al. (2010) [25]	Haihe River; 72-km matched water–sediment survey	Normalized sul1 and sul2 abundance correlated positively with summed sulfonamides across the river.	Cross-sectional association did not test decline after exposure.	Coupled cross-sectional association	M

Notes: M, Moderate; S, Suggestive. Evidence strength grades confidence in the observed paired response pattern. ARG, antibiotic resistance gene; MGE, mobile genetic element; WWTP, wastewater treatment plant.

Table S3. Representative quantitative evidence relevant to selected processes shown in the main-text mechanism schematic.

Process represented in the mechanism schematic	Study context	Representative quantitative evidence	Interpretive relevance	Source
Resuspension and phase partitioning	Simulated wind–wave disturbance in a water–sediment system	Suspended solids were 100, 500, and 1000 mg/L under increasing disturbance. Maximum sediment adsorption capacity reached 51.73 mg/kg, and norfloxacin accumulation in surface sediment was about 14-fold higher than sulfamethoxazole.	Quantifies compound-specific redistribution and sediment retention during resuspension; it does not by itself establish an ARG response.	[70]
Microplastic-associated ARG enrichment	Mariculture sediments exposed to polystyrene (PS) or polyvinyl chloride (PVC) microplastics of two size ranges	PS increased relative ARG abundance by 1.41–2.50-fold and PVC by 2.01–2.84-fold compared with the control.	Provides a quantitative example of microplastic-associated ARG enrichment and HGT-related processes under the tested conditions.	[71]
Transformation products and residual selection pressure	Escherichia coli exposed over time to tetracycline (TC) and the degradation products ATC, ETC, and EATC	Resistant-mutant frequency increased by up to 10^6-fold. In mixed exposure, TC, ATC, ETC, and EATC contributed 29.8%, 18.9%, 18.3%, and 31.9%, respectively, to resistance induction.	Shows that transformation products can retain resistance-selection relevance; the effect sizes are laboratory-specific.	[72]
Heavy-metal-associated co-selection	Low- and high-metal-polluted urban rivers in the UK and India	Network analysis showed positive MRG–ARG relationships with $r > 0.80$ and $p < 0.05$. Total or bioavailable metals were also strongly associated with relative MRG, ARG, and integron abundances ($\|r\| > 0.80$, $p < 0.05$).	Supports a strong field association consistent with metal-related co-selection, but the correlation is not a causal effect size.	[67]

Note: Values are retained as study-specific quantitative examples because the underlying designs, matrices, exposures, and endpoints are heterogeneous. They should not be interpreted as pooled effect estimates or universal selection thresholds. ARG, antibiotic resistance gene; HGT, horizontal gene transfer; MRG, metal resistance gene; PS, polystyrene; PVC, polyvinyl chloride.

Table S4. Comparative hydrodynamic settings, co-selective stressors, candidate mismatch mechanisms, and interpretive constraints across aquatic system types.

Aquatic system	Key hydrodynamic characteristics	Representative co-occurring or co-selective stressors	Candidate mismatch mechanism	Monitoring implication	Representative references
Rivers	Short residence time; continuous advection; frequent upstream–downstream exchange	Antibiotics, heavy metals, urban effluents	Continuous downstream reseeding; differential transport and retention	Longitudinal ARG signals may reflect continued loading or redistribution rather than legacy persistence	[26–36]
Lakes	Long residence time; stratification; weak horizontal mixing	Heavy metals, sulfide in anoxic layers, nutrients	Sediment retention, stratification, and seasonal redistribution	Seasonal stability or mismatch requires matched chemical–genetic time series	[6, 37–45]
Reservoirs	Artificial regulation; fluctuating water levels; periodic drawdown	Disinfectants, resuspension stress, metals	Drawdown and resuspension can remobilize stored material	Pulsed release creates exposure without proving in situ amplification	[46–49]
Estuaries	Hydrodynamic confinement; particle trapping (ETM); salinity gradients	Metals, microplastics, mixed pollutants	Particle trapping, mixed sources, and biofilm-associated transfer	Estuarine concentration and redistribution complicate source and legacy inference	[41–42, 50–55]
Aquaculture ponds	Low water exchange; high biomass density; internal recycling	Antibiotics, disinfectants, nutrients	Repeated loading and internal recycling	High ARG abundance may reflect continuing use and input rather than a post-pressure legacy	[56–59]
Urban water networks (WWTP-impacted rivers)	Engineered flow paths; frequent discharge pulses	Antibiotics, disinfectants, metals	Frequent discharge pulses and continuous organism or gene input	Downstream enrichment must be separated from treatment and loading dynamics	[60–65]

Note: ARG, antibiotic resistance gene; ETM, estuarine turbidity maximum; HGT, horizontal gene transfer; WWTP, wastewater treatment plant. This conceptual comparison links representative system characteristics to testable chemical–genetic monitoring strategies.

Table S5. Study-reported non-antibiotic stressor conditions associated with resistance-related responses.

Stressor	Reported exposure and duration	Matrix and design	Resistance-related response or effect size	Study context and monitoring implication	Source
Copper	50 or 100 μg L−1 for 20 d	River-water bacterial microcosms	Cefotaxime-resistant bacteria: 2.3% (control) to 9.5% (50 μg L−1) and 16.8% (100 μg L−1); tet-resistant bacteria: 0.03% to 0.23% at 100 μg L−1. No detectable increase in the assayed MGE-linked genes.	Supports short-term mechanistic selection under the tested conditions, without establishing a field threshold or persistent ARG enrichment.	[66]
Zinc	50 or 100 μg L−1 for 20 d	River-water bacterial microcosms	Cefotaxime-resistant bacteria: 24.6% (control) to 91.3% (50 μg L−1) and 72.4% (100 μg L−1); kanamycin-resistant bacteria: 6.1% to 24.1% and 43.0%, respectively.	Controlled short-term selection in river water; host-community change can explain the response.	[66]
Cadmium	No isolated exposure dose and duration in the retained aquatic field evidence	Urban-river water and sediment gradients with mixed metal and sewage sources	Total and bioavailable metal concentrations covaried with ARGs, metal-resistance genes, and integrons; the design did not isolate a cadmium-specific effect size.	Supports a mixed-source field association; cadmium-specific selection thresholds require source-resolved evidence.	[67]
Chlorine / disinfectant	0, 2, 6, or 10 mg L−1; duration is an SBR operating condition rather than a single comparable batch exposure	Activated-sludge SBRs; metagenomic and metatranscriptomic comparison	The low-dose condition increased ARG abundance, diversity, and transcriptional activity; higher doses reduced transcriptional diversity and activity.	Treatment-system evidence; field extrapolation requires measurement of residual exposure and receiving-water dilution.	[68]
UV-aged polystyrene microplastics	100 mg L−1 for 4 h	Activated sludge exposed to aged particles	Relative tetA and sul1 abundance increased approximately 6-fold and 1.6-fold, respectively, compared with the no-particle control.	High, short-term laboratory exposure; particle aging, leachate chemistry, and biomass constrain transfer to ambient waters.	[69]
Hydrogen sulfide	NR as a cross-study concentration–duration pair; study-specific redox conditions	Wastewater ecosystem experiment	Enhanced plasmid conjugation and broadened the recipient range of RP4 in wastewater microbiota.	Mechanistic evidence; ambient dissolved-sulfide exposure, host dynamics, and field transfer rates remain to be co-measured.	[61]

Note: Exposure units, matrices, histories, endpoints, and source contexts differ among studies. Values are retained to illustrate study-specific conditions rather than define cross-system selection thresholds. NR, no single comparable value.

References

[1] Garcia-Torné M, Falcó I, Borrell X, Bautista A, Mazigh R, Aznar R, Sánchez G, Farré M, Llorca M, 2025. Comprehensive Study of Antibiotics and Antibiotic Resistance Genes in Wastewater and Impacted Mediterranean Water Environments. Antibiotics 14, 341. https://doi.org/10.3390/antibiotics14040341

[2] Zhang L, Chen H, Gao S, Song Y, Zhao Y, Tang W, Cui J, 2024. Antibiotic resistance genes and mobile genetic elements in different rivers: The link with antibiotics, microbial communities, and human activities. Sci Total Environ 919, 170788. https://doi.org/10.1016/j.scitotenv.2024.170788

[3] Beltrán de Heredia I, Garbisu C, Alkorta I, Urra J, González-Gaya B, Ruiz-Romera E, 2023. Spatio-seasonal patterns of the impact of wastewater treatment plant effluents on antibiotic resistance in river sediments. Environ Pollut 319, 120883. https://doi.org/10.1016/j.envpol.2022.120883

[4] Gao FZ, He LY, Hu LX, Chen J, Yang YY, He LX, Bai H, Liu YS, Zhao JL, Ying GG, 2022. The variations of antibiotics and antibiotic resistance genes in two subtropical large river basins of south China: Anthropogenic impacts and environmental risks. Environ Pollut 312, 119978. https://doi.org/10.1016/j.envpol.2022.119978

[5] Li P, Wu Y, He Y, Zhang B, Huang Y, Yuan Q, Chen Y, 2020. Occurrence and fate of antibiotic residues and antibiotic resistance genes in a reservoir with ecological purification facilities for drinking water sources. Sci Total Environ 707, 135276. https://doi.org/10.1016/j.scitotenv.2019.135276

[6] Yuan J, Ni M, Liu M, Zheng Y, Gu Z, 2019. Occurrence of antibiotics and antibiotic resistance genes in a typical estuary aquaculture region of Hangzhou bay, China. Mar Pollut Bull 138, 376–384. https://doi.org/10.1016/j.marpolbul.2018.11.037

[7] Xu Y, Guo C, Lv J, Hou S, Luo Y, Zhang Y, Xu J, 2018. Spatiotemporal profile of tetracycline and sulfonamide and their resistance on a catchment scale. Environ Pollut 241, 1098-1105. https://doi.org/10.1016/j.envpol.2018.06.050

[8] Gao H, Li B, Li R, Lou Y, Jia X, Zhang S, Yao Z, 2026. Occurrence characteristics, cross-media behavior, and influencing factors of antibiotics and ARGs in typical rivers around the Bohai Sea. Mar Pollut Bull 226, 119322. https://doi.org/10.1016/j.marpolbul.2026.119322

[9] Ohore OE, Zhang J, Ifon BE, Kumwimba MN, Mu X, Kuang D, Wang Z, Gu JD, Yang G, 2024. Microbial phylogenetic divergence between surface-water and sedimentary ecosystems drove the resistome profiles. Sci Total Environ 915, 170122. https://doi.org/10.1016/j.scitotenv.2024.170122

[10] Wang L, Zhu M, Li Y, Zhao Z, Hu T, 2022. Deterministic assembly process dominates bacterial antibiotic resistome in wastewater effluents receiving river. Environ Sci Pollut Res 29, 90207-90218. https://doi.org/10.1007/s11356-022-22096-8

[11] Lekunberri I, Villagrasa M, Balcázar JL, Borrego CM, 2017. Contribution of bacteriophage and plasmid DNA to the mobilization of antibiotic resistance genes in a river receiving treated wastewater discharges. Sci Total Environ 601-602, 206-209. https://doi.org/10.1016/j.scitotenv.2017.05.174

[12] Berglund B, Fick J, Lindgren PE, 2015. Urban wastewater effluent increases antibiotic resistance gene concentrations in a receiving northern European river. Environ Toxicol Chem 34, 192-196. https://doi.org/10.1002/etc.2784

[13] Liang X, Guan F, Chen B, Luo P, Guo C, Wu G, Ye Y, Zhou Q, Fang H, 2020. Spatial and seasonal variations of antibiotic resistance genes and antibiotics in the surface waters of Poyang Lake in China. Ecotoxicol Environ Saf 196, 110543. https://doi.org/10.1016/j.ecoenv.2020.110543

[14] Wu T, Zhang Y, Wang B, Chen C, Cheng Z, Li Y, Wang B, Li J, 2022. Antibiotic resistance genes in Chishui River, a tributary of the Yangtze River, China: Occurrence, seasonal variation and its relationships with antibiotics, heavy metals and microbial communities. Sci Total Environ 846, 157472. https://doi.org/10.1016/j.scitotenv.2022.157472

[15] Jiang X, Liu L, Chen J, Fan X, Xie S, Huang J, Yu G, 2021. Antibiotic resistance genes and mobile genetic elements in a rural river in Southeast China: Occurrence, seasonal variation and association with the antibiotics. Sci Total Environ 778, 146131. https://doi.org/10.1016/j.scitotenv.2021.146131

[16] Zhang Y, Liu L, Liu Y, Chen L, Wang J, Li Y, Wang K, Wang W, 2024. Deciphering the natural and anthropogenic drivers on the fate and risk of antibiotics and antibiotic resistance genes (ARGs) in a typical river-estuary system, China. J Hazard Mater 480, 136006. https://doi.org/10.1016/j.jhazmat.2024.136006

[17] Lou S, Xiao C, Jin Y, Liu S, Dorzhievna RL, Fedorova IV, 2026. Occurrence of antibiotics and antibiotic resistance genes influenced by tides around a sewage outlet in the Yangtze Estuary, China. Mar Environ Res 214, 107766. https://doi.org/10.1016/j.marenvres.2025.107766

[18] Yang C, Yan S, Zhang B, Yao X, Mo J, Rehman F, Guo J, 2024. Spatiotemporal distribution of the planktonic microbiome and antibiotic resistance genes in a typical urban river contaminated by macrolide antibiotics. Environ Res 262, 119808. https://doi.org/10.1016/j.envres.2024.119808

[19] Chen A, Zhang T, Cheng F, Yang H, Guo Z, Zhao S, Zhang YN, Qu J, 2024. Comprehensive analysis and risk assessment of Antibiotic contaminants, antibiotic-resistant bacteria, and resistance genes: Patterns, drivers, and implications in the Songliao Basin. Environ Pollut 361, 124852. https://doi.org/10.1016/j.envpol.2024.124852

[20] Kerrigan JF, Sandberg KD, Engstrom DR, LaPara TM, Arnold WA, 2018. Small and large-scale distribution of four classes of antibiotics in sediment: association with metals and antibiotic resistance genes. Environ Sci Process Impacts 20, 1167-1179. https://doi.org/10.1039/c8em00190a

[21] Sabri NA, Schmitt H, Van der Zaan B, Gerritsen HW, Zuidema T, Rijnaarts HHM, Langenhoff AAM, 2020. Prevalence of antibiotics and antibiotic resistance genes in a wastewater effluent-receiving river in the Netherlands. J Environ Chem Eng 8, 102245. https://doi.org/10.1016/j.jece.2018.03.004

[22] Cho S, Hiott LM, Read QD, Damashek J, Westrich J, Edwards M, Seim RF, Glinski DA, Bateman McDonald JM, Ottesen EA, Lipp EK, Henderson WM, Jackson CR, Frye JG, 2023. Distribution of Antibiotic Resistance in a Mixed-Use Watershed and the Impact of Wastewater Treatment Plants on Antibiotic Resistance in Surface Water. Antibiotics 12, 1586. https://doi.org/10.3390/antibiotics12111586

[23] Tsholo K, Molale-Tom L, Horn S, Bezuidenhout CC, 2025. Bacterial community composition, quantification of antibiotic resistance genes and antibiotic residues in wastewater treatment plant and receiving rivers. Front Microbiol 16, 1635253. https://doi.org/10.3389/fmicb.2025.1635253

[24] Milaković M, Vestergaard G, González-Plaza JJ, Petrić I, Šimatović A, Senta I, Kublik S, Schloter M, Smalla K, Udiković-Kolić N, 2019. Pollution from azithromycin-manufacturing promotes macrolide-resistance gene propagation and induces spatial and seasonal bacterial community shifts in receiving river sediments. Environ Int 123, 501-511. https://doi.org/10.1016/j.envint.2018.12.050

[25] Luo Y, Mao D, Rysz M, Zhang H, Xu L, Alvarez PJJ, 2010. Trends in antibiotic resistance genes occurrence in the haihe river, china. Environ Sci Technol 44, 7220–7225. https://doi.org/10.1021/es100233w

[26] Garner E, Benitez R, Wagoner E von, Sawyer R, Schaberg E, Hession WC, Krometis L-AH, Badgley BD, Pruden A, 2017. Stormwater loadings of antibiotic resistance genes in an urban stream. Water Res 123, 144–152. https://doi.org/10.1016/j.watres.2017.06.046

[27] Yin Z, 2021. Distribution and ecological risk assessment of typical antibiotics in the surface waters of seven major rivers, China. Environ Sci: Processes Impacts 23, 1088–1100. https://doi.org/10.1039/D1EM00079A

[28] Liu W, Zhou C, Wang X, Bai X, Ren Y, 2024. Spatiotemporal distribution of ecological risk of antibiotics in seven major river basins of China: an optimized multilevel assessment approach. Water Sci Technol 89, 2035–2043. https://doi.org/10.2166/wst.2024.100

[29] Dong J, Shang M, Feng R, Song X, Yan D, Xie H, 2022. Export and risk from antibiotic remobilization from surrounding water to lake in the extreme 2020 yangtze river basin flooding. Sci Total Environ 834, 155176. https://doi.org/10.1016/j.scitotenv.2022.155176

[30] Liu X-Y, Gbadegesin LA, He Y, Zhang J-Q, Liu C, 2025. Plot-scale observation on antibiotics migration in surface runoff and leachate from chicken-raising orchard of entisol during rainstorms. Ecotoxicol Environ Saf 294, 118105. https://doi.org/10.1016/j.ecoenv.2025.118105

[31] Lai C, Wang Z, Gu T, Jian L, Meng X, Meng Q, Gao D, 2025. Overall evaluation of antibiotics occurrence from large-scale livestock farms in sichuan basin, china: spatial distribution, source apportionment, and risk assessment. Toxics 13, 154. https://doi.org/10.3390/toxics13030154

[32] Wang D, Li M, Liao C, Dong K, Yao Y, 2025. The occurrence characteristics and ecological risks of antibiotics in surface water and groundwater of the Huixian Wetland. Toxics 13. https://doi.org/10.3390/toxics13060460

[33] Barrantes-Jiménez K, Mendoza-Guido B, Morales-Mora E, Rivera-Montero L, Montiel-Mora J, Chacón-Jiménez L, Rojas-Jiménez K, Arias-Andrés M, 2025. Influence of Seasonality and Pollution on the Presence of Antibiotic Resistance Genes and Potentially Pathogenic Bacteria in a Tropical Urban River. Antibiotics 14, 798. https://doi.org/10.3390/antibiotics14080798

[34] Javvadi Y, Mohan SV, 2024. Temporal dynamics and persistence of resistance genes to broad spectrum antibiotics in an urban community. npj Clean Water 7, 56. https://doi.org/10.1038/s41545-024-00349-y

[35] Hou L, Li J, Wang H, Chen Q, Su J-Q, Gad M, Ahmed W, Yu C-P, Hu A, 2022. Storm promotes the dissemination of antibiotic resistome in an urban lagoon through enhancing bio-interactions. Environ Int 168, 107457. https://doi.org/10.1016/j.envint.2022.107457

[36] Shang M, Dong J, Xie H, Li Y, Wan N, Xiong Z, Lai X, 2025. Unraveling transport mechanisms and rainfall event-driven controls on non-point source antibiotic pollution by high-resolution process-based modeling. J Hazard Mater 495, 139177. https://doi.org/10.1016/j.jhazmat.2025.139177

[37] Liu X, Lu S, Guo W, Xi B, Wang W, 2018. Antibiotics in the aquatic environments: a review of lakes, China. Sci Total Environ 627, 1195–1208. https://doi.org/10.1016/j.scitotenv.2018.01.271

[38] Wei C, Wang Y, Zhang R, Liu F, Zhang Z-E, Wang J, Yu K, 2024. Spatiotemporal distribution and potential risks of antibiotics in coastal water of beibu gulf, south China sea: Livestock and poultry emissions play essential effect. J Hazard Mater 466, 133550. https://doi.org/10.1016/j.jhazmat.2024.133550

[39] Lu S, Lin C, Lei K, Xin M, Gu X, Lian M, Wang B, Liu X, Ouyang W, He M, 2022. Profiling of the spatiotemporal distribution, risks, and prioritization of antibiotics in the waters of Laizhou bay, northern China. J Hazard Mater 424, 127487. https://doi.org/10.1016/j.jhazmat.2021.127487

[40] Wang J, Hao R, He X, Chen J, Wang H, 2026. Coastal antibiotics hotspots: decoding spatiotemporal drivers and risk gradients in Chinese marine ecoregions. Mar Pollut Bull 223, 118965. https://doi.org/10.1016/j.marpolbul.2025.118965

[41] Zhao Z, Zhang K, Wu N, Li W, Xu W, Zhang Y, Niu Z, 2020. Estuarine sediments are key hotspots of intracellular and extracellular antibiotic resistance genes: a high-throughput analysis in haihe estuary in China. Environ Int 135, 105385. https://doi.org/10.1016/j.envint.2019.105385

[42] Wu C, Zhang G, Xu W, Jian S, Peng L, Jia D, Sun J, 2021. New estimation of antibiotic resistance genes in sediment along the haihe river and bohai bay in China: a comparison between single and successive DNA extraction methods. Front Microbiol 12. https://doi.org/10.3389/fmicb.2021.705724

[43] Zhang R, Zhang R, Zou S, Yang Y, Li J, Wang Y, Yu K, Zhang G, 2017. Occurrence, distribution and ecological risks of fluoroquinolone antibiotics in the dongjiang river and the beijiang river, pearl river delta, south China. Bull Environ Contam Toxicol 99, 46–53. https://doi.org/10.1007/s00128-017-2107-5

[44] Hsu L-C, Liu Y-T, Syu C-H, Huang M-H, Tzou Y-M, Teah HY, 2018. Adsorption of tetracycline on Fe (hydr)oxides: effects of pH and metal cation (Cu2+, Zn2+ and Al3+) addition in various molar ratios. R Soc Open Sci 5, 171941. https://doi.org/10.1098/rsos.171941

[45] Ye F, Chu K, Hua Z, Liu Y, Yang X, Qing S, Gu L, 2025. Distribution and fate of antibiotics in water–sediment–biofilm phases and their potential risks in urban lakes within a large-scale city in eastern China. J Hazard Mater 496, 139421. https://doi.org/10.1016/j.jhazmat.2025.139421

[46] Tong X, Goh SG, Mohapatra S, Tran NH, You L, Zhang J, He Y, Gin KY-H, 2024. Predicting antibiotic resistance and assessing the risk burden from antibiotics: a holistic modeling framework in a tropical reservoir. Environ Sci Technol 58, 6781–6792. https://doi.org/10.1021/acs.est.3c10467

[47] Li S, Zhang Q, Chen M, Strokal M, Kroeze C, Ma L, Zhang F, Chen X, Chen X, Hofstra N, 2025. Antibiotics sources, concentrations, and risk of antimicrobial resistance selection in the three gorges reservoir basin, China. Environ Sci Technol 59, 19398–19413. https://doi.org/10.1021/acs.est.5c00740

[48] Huang L, Mo Y, Wu Z, Rad S, Song X, Zeng H, Bashir S, Kang B, Chen Z, 2020. Occurrence, distribution, and health risk assessment of quinolone antibiotics in water, sediment, and fish species of Qingshitan reservoir, south China. Sci Rep 10, 15777. https://doi.org/10.1038/s41598-020-72324-9

[49] Huang X, Wang C, Chen Y, Zhang M, Hashmi MZ, Sun T, Zou B, Zhang Y, Lin J, Wang Z, 2025. Seasonal water level fluctuations regulate the source, distribution, and risk of antibiotics in the largest floodplain-lake in China. Water Res 286, 124158. https://doi.org/10.1016/j.watres.2025.124158

[50] Leng Y, Xiao H, Li Z, Wang J, 2020. Tetracyclines, sulfonamides and quinolones and their corresponding resistance genes in coastal areas of Beibu gulf, China. Sci Total Environ 714, 136899. https://doi.org/10.1016/j.scitotenv.2020.136899

[51] Zheng C-L, Ruan T, Chan FKS, Bao P, Li G, Xu Y-Y, 2021. Statistical approach reveals tidal effect on the antibiotics and environmental relationship with the case study of Yongjiang estuary, China. Mar Environ Res 164, 105244. https://doi.org/10.1016/j.marenvres.2020.105244

[52] Li F, Mai Z, Qiu C, Long L, Hu A, Huang S, 2023. Dissemination of antibiotic resistance genes from the pearl river estuary to adjacent coastal areas. Mar Environ Res 188, 105978. https://doi.org/10.1016/j.marenvres.2023.105978

[53] He Y, Bai M, He Y, Wang S, Zhang J, Jiang S, Wang G, 2022. Suspended particles are hotspots for pathogen-related bacteria and ARGs in coastal beach waters of northern China. Sci Total Environ 817, 153004. https://doi.org/10.1016/j.scitotenv.2022.153004

[54] Wang K, Su Z, Reguyal F, Bian R, Li W, Yu H, Sun Y, Zhuang Y, Shang W, 2022. Seasonal occurrence of multiple classes of antibiotics in east China rivers and their association with suspended particulate matter. Sci Total Environ 853, 158579. https://doi.org/10.1016/j.scitotenv.2022.158579

[55] Wen L, Dai J, Ma J, Li X, Yuan H, Duan L, Zhao C, Song J, 2025. Comprehensive profiling of quinolone antibiotics in the bohai sea: occurrence, source apportionment, and environmental risks. Environ Pollut 387, 127338. https://doi.org/10.1016/j.envpol.2025.127338

[56] Han QF, Zhang XR, Xu XY, Wang XL, Yuan XZ, Ding ZJ, Zhao S, Wang SG, 2021. Antibiotics in marine aquaculture farms surrounding laizhou bay, bohai sea: distribution characteristics considering various culture modes and organism species. Sci Total Environ 760, 143863. https://doi.org/10.1016/j.scitotenv.2020.143863

[57] Jie Zhao, Yuze Han, Junzhao Liu, Baoju Li, Jun Li, Wentao Li, Peng Shi, Yang Pan, Aimin Li, 2024. Occurrence, distribution and potential environmental risks of pollutants in aquaculture ponds during pond cleaning in Taihu Lake Basin, China. Sci Total Environ 939, 173610. https://doi.org/10.1016/j.scitotenv.2024.173610

[58] Chen L, Shi J, Wu D, Zhu Y, Adams JM, Wu J, Chen X, Guo H, 2025. Unveiling the role of wetland strategies in antibiotic risk reduction across China by machine learning. Environ Sci Technol 59, 15865–15876. https://doi.org/10.1021/acs.est.5c02866

[59] Wu C, Ye H, Xu M, Zhao X, Zhao X, Li L, Li M, Wei Y, Li Y, Hu B, 2025. Occurrence of antibiotics and antibiotic resistance genes at various stages of different aquaculture modes surrounding tai lake, china. Front Microbiol 16. https://doi.org/10.3389/fmicb.2025.1543387

[60] Larsson DGJ, Flach C-F, 2021. Antibiotic resistance in the environment. Nat Rev Microbiol 20, 257. https://doi.org/10.1038/s41579-021-00649-x

[61] Huang H, Lin L, Liu Q, Li X, Liao J, Dong L, Zhang J, Su Y, Luo J, Feng L, Zheng X, Guo J, Chen Y, 2025. Hydrogen sulfide drives horizontal transfer of plasmid-borne antibiotic resistance genes in wastewater ecosystems. Nat Water. https://doi.org/10.1038/s44221-025-00523-7

[62] Balta I, Lemon J, Gadaj A, Cretescu I, Stef D, Pet I, Stef L, McCleery D, Douglas A, Corcionivoschi N, 2025. The interplay between antimicrobial resistance, heavy metal pollution, and the role of microplastics. Front Microbiol 16. https://doi.org/10.3389/fmicb.2025.1550587

[63] Wales AD, Davies RH, 2015. Co-selection of resistance to antibiotics, biocides and heavy metals, and its relevance to foodborne pathogens. Antibiotics 4, 567–604. https://doi.org/10.3390/antibiotics4040567

[64] Murray AK, Stanton IC, Tipper HJ, Wilkinson H, Schmidt W, Hart A, Singer AC, Gaze WH, 2024. A critical meta-analysis of predicted no effect concentrations for antimicrobial resistance selection in the environment. Water Res 266, 122310. https://doi.org/10.1016/j.watres.2024.122310

[65] Fernanda PA, Liu S, Yuan T, Ramalingam B, Lu J, Sekar R, 2022. Diversity and abundance of antibiotic resistance genes and their relationship with nutrients and land use of the inflow rivers of Taihu Lake. Front Microbiol 13, 1009297. https://doi.org/10.3389/fmicb.2022.1009297

[66] Silva I, Tacão M, Henriques I, 2021. Selection of antibiotic resistance by metals in a riverine bacterial community. Chemosphere 263, 127936. https://doi.org/10.1016/j.chemosphere.2020.127936

[67] Gupta S, Graham DW, Sreekrishnan TR, Ahammad SZ, 2022. Effects of heavy metals pollution on the co-selection of metal and antibiotic resistance in urban rivers in UK and India. Environ Pollut 306, 119326. https://doi.org/10.1016/j.envpol.2022.119326

[68] Meng Q, Wang J, Li K, Zhang Y, Hu Z, Wang F, Pan F, Fu J, Dang C, 2026. Low-dose chlorine disinfection poses a greater potential risk of antibiotic resistance genes and their pathogenic hosts. Water Res 289, 124895. https://doi.org/10.1016/j.watres.2025.124895

[69] Yuan Q, Sun R, Yu P, Cheng Y, Wu W, Bao J, Alvarez PJJ, 2022. UV-aging of microplastics increases proximal ARG donor-recipient adsorption and leaching of chemicals that synergistically enhance antibiotic resistance propagation. Journal of Hazardous Materials 427, 127895. https://doi.org/10.1016/j.jhazmat.2021.127895

[70] Li S, Huang Z, Wang Y, Liu Y-Q, Luo R, Shang J-G, Liao Q-J-H, 2018. Migration of two antibiotics during resuspension under simulated wind–wave disturbances in a water–sediment system. Chemosphere 192, 234–243. https://doi.org/10.1016/j.chemosphere.2017.10.131

[71] Liu Y, Liu L, Wang X, Shao M, Wei Z, Wang L, Li B, Li C, Luo X, Li F, Zheng H, 2025. Microplastics enhance the prevalence of antibiotic resistance genes in mariculture sediments by enriching host bacteria and promoting horizontal gene transfer. Eco-Environ Health 4, 100136. https://doi.org/10.1016/j.eehl.2025.100136

[72] Guo Z, Zhang T, Yang H, Zhu X, Lu S, Chen A, Fan M, Qu J, 2025. Unraveling tetracycline and its degradation product: Induction mechanisms of antibiotic resistance in Escherichia coli. Sci Total Environ 970, 178959. https://doi.org/10.1016/j.scitotenv.2025.178959
